# Genetic overlap and causal associations between smoking behaviours and mental health

**DOI:** 10.1038/s41598-021-93962-7

**Published:** 2021-07-21

**Authors:** Wikus Barkhuizen, Frank Dudbridge, Angelica Ronald

**Affiliations:** 1 Centre for Brain and Cognitive Development, Department of Psychological Sciences, Birkbeck University of London, Malet Street, London, WC1E 7HX UK; 2 Department of Clinical, Educational and Health Psychology, University College London, London, UK; 3 Department of Health Sciences, University of Leicester, Leicester, UK

**Keywords:** Structural variation, Human behaviour, Development

## Abstract

Cigarette smoking is a modifiable behaviour associated with mental health. We investigated the degree of genetic overlap between smoking behaviours and psychiatric traits and disorders, and whether genetic associations exist beyond genetic influences shared with confounding variables (cannabis and alcohol use, risk-taking and insomnia). Second, we investigated the presence of causal associations between smoking initiation and psychiatric traits and disorders. We found significant genetic correlations between smoking and psychiatric disorders and adult psychotic experiences. When genetic influences on known covariates were controlled for, genetic associations between most smoking behaviours and schizophrenia and depression endured (but not with bipolar disorder or most psychotic experiences). Mendelian randomization results supported a causal role of smoking initiation on psychiatric disorders and adolescent cognitive and negative psychotic experiences, although not consistently across all sensitivity analyses. In conclusion, smoking and psychiatric disorders share genetic influences that cannot be attributed to covariates such as risk-taking, insomnia or other substance use. As such, there may be some common genetic pathways underlying smoking and psychiatric disorders. In addition, smoking may play a causal role in vulnerability for mental illness.

## Introduction

Tobacco smoking is a modifiable risk factor and despite declining rates of smoking, 14–15% of adults in the US and UK and 28% across Europe still smoke regularly^1–3^. High co-occurrence between smoking behaviours and psychiatric disorders is well-established^4–6^. Smoking rates among individuals diagnosed with schizophrenia or bipolar disorder are five times greater and with depression two-fold greater compared to healthy controls^7,8^. Smoking behaviours also co-occur with psychotic experiences^9–11^, traits in the general population that typically resemble positive symptoms of psychotic disorders (e.g., paranoia, hallucinations and delusions). Smoking behaviours co-occur with schizotypy^12^, a construct that has parallels with psychotic experiences and which assesses personality characteristics thought to reflect schizophrenia vulnerability. Regular smoking during adolescence has been associated with psychotic experiences and negative symptom traits (PENS) such as anhedonia^13^.

In terms of underlying causes, shared genetic influences could explain the co-occurrence of smoking and psychiatric traits or disorders. A recent twin study found that regular smoking during adolescence shared genetic influences with paranoia and cognitive disorganization and familial influences with hallucinations^13^. A previous study found no evidence that adolescent PENS were predicted by polygenic liability to initiate smoking^14^ but used less well-powered genome-wide association study (GWAS) summary statistics than currently available. Schizophrenia and major depression share genome-wide genetic influences with smoking behaviours^15,16^, and polygenic liability to bipolar disorder has been associated with nicotine dependence^17^. Genetic variants for smoking being identified in GWAS for schizophrenia could also indicate possible causal associations between these traits^18^.

Several covariates could, at a genetic level, account for genetic overlap between psychiatric traits or disorders and smoking behaviour. Epidemiological studies have investigated cannabis and alcohol use, stressful or traumatic events, sociodemographic characteristics, novelty-seeking behaviour and sleep disturbances as covariates in the association between smoking and mental health problems^9–11,13,19–23^. Risk-taking has also been associated with smoking^24,25^ and psychiatric outcomes^26^. Many of these covariates, including cannabis and alcohol use, risk-taking and insomnia, are partly under genetic influence and have been genetically associated with smoking or with psychiatric traits or disorders^27–32^.

Smoking behaviour may be a causal risk factor for psychiatric disorders based on evidence from longitudinal and dose–response associations and Mendelian randomization studies^19,21,23,33,34^. Uncertainty remains over whether smoking may be causally linked to psychotic experiences prior to the onset of psychiatric conditions or in individuals who may not meet diagnostic thresholds: The association between psychotic experiences and smoking is not fully explained by known confounding factors^9,35–39^, (but see^35^) and a dose–response relationship has been reported^9,40^ although not consistently^35,38^. Furthermore, most longitudinal studies report an association between smoking and later reports of psychotic experiences^10,41–43^ while adolescents do not appear to start smoking to alleviate pre-existing psychotic experiences^41^. However, these observational studies cannot rule out the possibility that psychotic experiences were present prior to smoking initiation and vice-versa. Triangulation of longitudinal findings on the association between smoking and psychotic experiences with evidence from methods such as Mendelian randomization is needed.

In this study we employed a range of methods utilizing genetic data to systematically investigate the association between smoking behaviours and psychotic experiences across adolescence and adulthood as well as with psychiatric disorders. We assessed the degree to which smoking behaviours are genetically correlated with major psychiatric disorders (schizophrenia, major depression and bipolar disorder), with psychotic experiences during adolescence (paranoia and hallucinations, cognitive disorganization, anhedonia and negative symptoms), psychotic experiences in adults (auditory hallucinations, visual hallucinations, delusions of persecution and delusions of reference), and with schizotypy in adults (hypomania, perceptual aberrations, physical anhedonia and social anhedonia) after controlling for genetic overlap with cannabis and alcohol use, risk taking behaviour and sleep disturbances^27–31^. Furthermore, we aimed to assess causal associations between smoking initiation and psychotic experiences across development and confirm previous reports of causal associations with psychiatric disorders.

## Results

### Conditional genetic overlap between smoking behaviours and mental health

**Figure 1:**
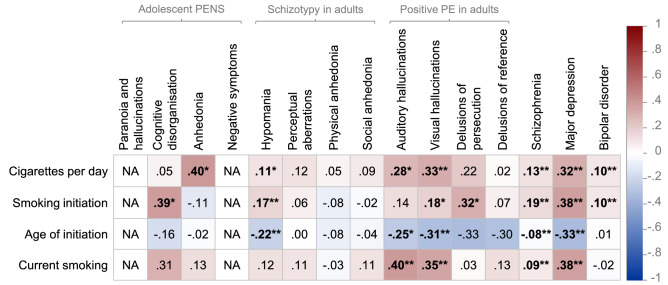
Heat map showing genetic correlations between smoking behaviours, psychotic experiences and psychiatric disorders. *PENS* Psychotic experiences (PE) and negative symptom traits; *NA* indicates that genetic correlations could not be computed due to low SNP-heritability or sample size; *indicates statistically significant genetic correlations at *p* < .05; **indicates significance at FDR < .05 using Benjamini–Hochberg correction for 60 tests; Genetic correlations reported using unconstrained LD score regression intercept between phenotypes with sample overlap (for example, smoking behaviour and major depression GWASs contained participants from the UK Biobank, and smoking behaviours and adolescent PENS contained participants from ALSPAC). Note that age of smoking initiation was coded in the direction of lower scores reflecting younger ages of initiation. Note that current smoking cases are current smokers (compared to ex-smokers).

Bivariate genetic correlations between smoking behaviours and psychotic experiences and psychiatric disorders are summarised in Fig. 1.

To investigate the degree to which these genetic correlations exist beyond genetic influences associated with covariates (lifetime cannabis use, alcohol consumption, insomnia and risk-taking behaviour), we specified genomic multiple regression models using Genomic Structural Equation Modelling (Genomic SEM)^44^. Models were run for phenotype pairs that shared at least nominally significant (*p* < 0.05) genetic overlap. Genetic correlations between smoking behaviours and the covariates are shown in Supplementary Figure S1.

**Figure 2:**
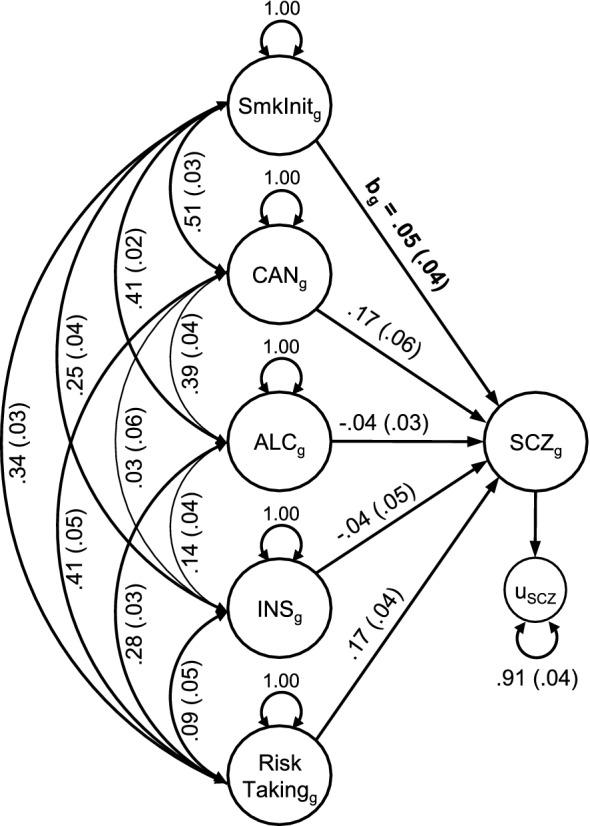
Path diagram illustrating genetic multiple regression models. SmkInit_g_ = Genetic component of smoking initiation; CAN_g_ = Genetic component of cannabis use; ALC_g_ = Genetic component of alcohol consumption; INS_g_ = Genetic component of insomnia; SCZ_g_ = Genetic component of schizophrenia; u = residual genetic variance; b_g_ = Conditional genetic correlation between the genetic components of smoking initiation and schizophrenia as summarized in Table 1; Double-headed arrow represents genetic covariance; Single-headed arrow represents regression paths; Path diagrams for models between all smoking behaviours and psychiatric phenotypes are provided in Supplementary Figures S2-5.

Figure 2 shows the path diagram between schizophrenia and smoking initiation to illustrate the specified covariance paths in the models (see Supplementary Figures S2-5 for path diagrams of all models). Table 1 summarizes the conditional genetic correlations (*b*_g_) between smoking behaviours and psychiatric disorders/psychotic experiences obtained from these models.

**Table 1:** Conditional genetic correlations after accounting for genetic influences on covariates in genomic structural equation models.

Psychiatric disorders	Smoking behaviours	LD score regression			Standardized conditional genetic associations		
		r_g_	SE	*p*	b_g_	SE	*p*
Schizophrenia	Cigarettes per day	0.13	0.02	2.27 × 10^–11^	0.14	0.04	**6.81 × 10**^**–4**^
Schizophrenia	Smoking initiation	0.19	0.02	2.69 × 10^–31^	0.05	0.04	0.209
Schizophrenia	Age of smoking initiation	− 0.08	0.02	1.97 × 10^–5^	− 0.03	0.04	0.434
Schizophrenia	Current smoking	0.09	0.02	1.00 × 10^–4^	0.16	0.05	**8.17 × 10**^**–4**^
Major depression	Cigarettes per day	0.32	0.05	6.15 × 10^–12^	0.17	0.06	**0.003**
Major depression	Smoking initiation	0.38	0.03	1.57 × 10^–29^	0.16	0.07	**0.017**
Major depression	Age of smoking initiation	− 0.33	0.04	1.16 × 10^–14^	− 0.11	0.06	0.075
Major depression	Current smoking	0.38	0.05	1.31 × 10^–14^	0.30	0.07	**1.93 × 10**^**–5**^
Bipolar disorder	Cigarettes per day	0.10	0.04	0.006	0.05	0.05	0.263
Bipolar disorder	Smoking initiation	0.10	0.03	7.00 × 10^–4^	− 0.11	0.05	**0.029**
**Adult PE**							
Auditory hallucinations	Cigarettes per day	0.28	0.12	0.015	0.05	0.12	0.662
Auditory hallucinations	Age of smoking initiation	− 0.25	0.12	0.037	0.00	0.12	0.999
Auditory hallucinations	Current smoking	0.40	0.15	0.009	0.21	0.14	0.125
Visual hallucinations	Cigarettes per day	0.33	0.09	4.00 × 10^–4^	0.18	0.13	0.165
Visual hallucinations	Smoking initiation	0.18	0.08	0.026	0.14	0.12	0.266
Visual hallucinations	Age of smoking initiation	− 0.31	0.10	0.002	− 0.15	0.13	0.248
Visual hallucinations	Current smoking	0.35	0.11	0.001	0.26	0.13	**0.044**
Delusions of persecution	Smoking initiation	0.32	0.13	0.015	0.20	0.18	0.258
**Schizotypy**							
Hypomania	Cigarettes per day	0.11	0.06	0.049	− 0.01	0.17	0.968
Hypomania	Smoking initiation	0.17	0.05	8.00 × 10^–4^	0.00	0.16	0.980
Hypomania	Age of smoking initiation	− 0.22	0.07	0.002	− 0.04	0.16	0.820
**Adolescent PENS**							
Anhedonia	Cigarettes per day	0.40	0.20	0.046	0.44	0.22	**0.044**
Cognitive disorganization	Smoking initiation	0.39	0.16	0.017	0.09	0.22	0.676

r_g_ = Bivariate genetic correlation estimates calculated in LD score regression; b_g_ = Conditional genetic association estimates (accounting for genetic influences on cannabis and alcohol use, insomnia and risk taking) obtained from Genomic Structural Equasion Modelling that, in standardized form, can be interpreted as conditional genetic correlations accounting for genetic covariation between predictors and regression paths of covariates onto psychiatric outcomes; PE = Psychotic experiences; PENS = Psychotic experiences and negative symptom traits; *p* value thresholds for b_g_ set at 0.05 and displayed in bold text.

The genetic component of cigarettes per day, together with the genetic components of the four covariate predictors, accounted for 11–41% of genetic variation in psychiatric disorders and traits (calculated as one minus the residual variance shown in the figures). The equivalent values for smoking initiation, age of smoking initiation and current smoking status were 9–52%, 9–41%, and 11–41%, respectively.

Table 1 shows that after accounting for the genetic influences of the covariates, conditional genetic associations (b_g_) were significant between cigarettes per day and schizophrenia (unattenuated compared to r_g_ estimates that did not account for covariates), depression (with b_g_ accounting for 53% of r_g_; calculated as b_g_/r_g_ × 100) and adolescent anhedonia (unattenuated) but not with bipolar disorder, auditory hallucinations, visual hallucinations and hypomania.

Significant conditional genetic associations were found between smoking initiation and depression, accounting for 42% of the r_g_ estimate from LD score regression. The conditional genetic association between smoking initiation and bipolar disorder was negative. This suggests that overlapping genetic influences of the covariates drove the positive genetic correlation observed in LD score regression. No genetic association was found between smoking initiation and schizophrenia, visual hallucinations, delusions of persecution, hypomania and adolescent cognitive disorganization after accounting for the genetic influences of covariates.

No significant conditional genetic associations were found between age of smoking initiation with schizophrenia, depression, auditory hallucinations, visual hallucinations and hypomania. Negative conditional genetic association estimates indicate an association with a younger age of smoking initiation.

Significant genetic overlap not accounted for by other predictors in the model was found between current smoking and schizophrenia (with b_g_ being higher than the r_g_ estimate from LD score regression), depression (accounting for 79% of r_g_) and visual hallucinations (74% of r_g_) and not with auditory hallucinations. In summary, after controlling for genetic overlap with cannabis and alcohol use, insomnia and risk-taking behaviour, most smoking behaviours still showed significant genetic overlap with psychiatric disorders, but not with most measures of psychotic experiences.

### Causal associations between smoking initiation and mental health

Table 2 presents the results from Mendelian randomization (MR; see also Supplementary Figures S6-13 and Supplementary Tables S2-4). All instrumental variables had a mean F > 10 indicating adequate strength of association with the exposures, and an I^2^ > 0.9 and therefore MR-Egger sensitivity analyses can be considered reliable. Generalized summary-based Mendelian randomization (GSMR)^45^ and inverse-variance weighted Mendelian randomization (MR-IVW)^46^ analyses yielded consistent results across analyses. These methods suggested a causal effect of smoking initiation on schizophrenia liability with this finding replicated in Weighted Median but not in MR-Egger or Weighted Mode sensitivity analyses, suggesting that results may have been biased due to violation of the instrumental variable assumptions^47–49^. A significant but smaller causal effect of schizophrenia liability on smoking initiation was also found, but the MR-Egger intercept was significantly different from zero (*p* = 0.043; Supplementary Table S2), indicating the presence of directional pleiotropy which could bias results from GSMR.

**Table 2:** Mendelian randomization results.

Exposure	Outcome	n SNP	MR-IVW results			GSMR results			MR-Egger			Weighted median			Weighted mode		
			Beta	95% *CI*	*p*	Beta	95% *CI*	*p*	Beta	95% *CI*	*p*	Beta	95% *CI*	*p*	Beta	95% *CI*	*p*
**Smoking and psychiatric disorders**																	
Smoking initiation	Schizophrenia	92	0.665	0.315; 1.016	**1.99 × 10**^**–4**^	0.579	0.371; 0.788	**5.33 × 10**^**–8**^	0.305	− 1.523; 2.134	0.744	0.385	0.031; 0.740	**0.033**	0.067	− 0.992; 1.126	0.901
Schizophrenia	Smoking initiation	125	0.017	0.009;0.024	**1.35 × 10**^**–5**^	0.015	0.010; 0.020	**1.18 × 10**^**–8**^	0.047	0.017; 0.078	**0.003**	0.019	0.010; 0.028	**4.82 × 10**^**–6**^	0.034	0.003; 0.066	**0.036**
Smoking initiation^a^	Major depression	20	0.434	0.238; 0.630	**1.39 × 10**^**–5**^	0.365	0.248; 0.482	**8.99 × 10**^**–10**^	− 0.845	− 2.041; 0.351	0.183	0.324	0.128; 0.519	**0.001**	0.061	− 0.296; 0.418	0.771
Major depression	Smoking initiation^a^	29	− 0.000	− 0.003; 0.003	0.930	− 0.000	− 0.002; 0.002	0.896	0.135	0.047; 0.224	**0.006**	− 0.001	− 0.004; 0.003	0.642	− 0.003	− 0.010; 0.005	0.449
Smoking initiation	Bipolar disorder	106	0.902	0.520; 1.283	**3.61 × 10**^**–6**^	0.812	0.515; 1.108	**7.82 × 10**^**–8**^	0.795	− 1.076; 2.666	0.407	0.800	0.341; 1.259	**0.001**	0.893	− 0.237; 2.023	0.124
Bipolar disorder	Smoking initiation	18	0.008	− 0.011; 0.027	0.415	0.006	− 0.007; 0.019	0.356	− 0.098	− 0.213; 0.016	0.112	− 0.003	− 0.023; 0.017	0.767	− 0.009	− 0.042; 0.023	0.578
**Smoking and positive psychotic experiences in adults**																	
Smoking initiation^a^	Auditory hallucinations	21	0.004	− 0.006; 0.014	0.447	0.004	− 0.004; 0.011	0.323	− 0.012	− 0.069; 0.045	0.680	0.001	− 0.010; 0.013	0.813	− 0.004	− 0.023; 0.016	0.709
Auditory hallucinations	Smoking initiation^a^	90	− 0.114	− 0.645; 0.418	0.675	− 0.119	− 0.637; 0.398	0.651	0.623	− 0.547; 1.793	0.299	− 0.242	− 0.997; 0.513	0.529	− 0.432	− 2.362; 1.497	0.662
Smoking initiation^a^	Visual hallucinations	21	0.001	− 0.009; 0.011	0.859	0.001	− 0.009; 0.011	0.820	− 0.023	− 0.079; 0.033	0.423	− 0.002	− 0.016; 0.012	0.805	− 0.004	− 0.032; 0.025	0.798
Visual hallucinations	Smoking initiation^a^	81	0.217	− 0.206; 0.640	0.314	0.197	− 0.212; 0.605	0.345	0.499	− 0.496; 1.495	0.329	0.192	− 0.388; 0.771	0.517	0.175	− 1.187; 1.538	0.802
Smoking initiation^a^	Delusions of reference	21	0.003	− 0.002; 0.007	0.276	0.003	− 0.002; 0.007	0.280	0.009	− 0.019; 0.036	0.543	0.001	− 0.005; 0.008	0.669	− 0.005	− 0.020; 0.010	0.545
Delusions of reference	Smoking initiation^a^	105	0.003	− 0.725; 0.731	0.994	− 0.042	− 0.801; 0.717	0.914	− 0.449	− 1.951; 1.054	0.560	− 0.234	− 1.316; 0.848	0.672	0.392	− 2.565; 3.349	0.795
Smoking initiation^a^	Delusions of persecution	21	0.003	− 0.001; 0.008	0.168	0.003	− 0.002; 0.008	0.193	− 0.003	− 0.031; 0.025	0.820	0.003	− 0.004; 0.009	0.441	0.002	− 0.011; 0.015	0.807
Delusions of persecution	Smoking initiation^a^	94	0.154	− 0.657; 0.964	0.710	0.149	− 0.608; 0.907	0.700	− 0.044	− 1.756; 1.670	0.960	− 0.324	− 1.383; 0.736	0.549	− 1.034	− 3.733; 1.664	0.454
**Smoking and schizotypy**																	
Smoking initiation	Hypomania	98	0.241	− 0.240; 0.723	0.326	0.235	− 0.224; 0.695	0.316	0.069	− 2.125; 2.264	0.951	0.111	− 0.554; 0.775	0.744	− 0.040	− 1.653; 1.573	0.961
Hypomania	Smoking initiation	68	− 0.001	− 0.006; 0.004	0.644	− 0.001	− 0.005; 0.003	0.565	− 0.004	− 0.014; 0.007	0.494	− 0.002	− 0.008; 0.004	0.455	− 0.003	− 0.015; 0.009	0.609
Smoking initiation	Perceptual aberrations	98	0.065	− 0.438; 0.568	0.800	0.036	− 0.412; 0.490	0.878	0.591	− 1.699; 2.881	0.614	0.154	− 0.502; 0.810	0.645	0.744	− 0.740; 2.227	0.328
Perceptual aberrations	Smoking initiation	54	− 0.001	− 0.005; 0.004	0.731	− 0.000	− 0.005; 0.004	0.867	− 0.001	− 0.011; 0.008	0.792	− 0.001	− 0.008; 0.006	0.729	0.000	− 0.011; 0.010	0.951
Smoking initiation	Physical anhedonia	98	− 0.166	− 0.684; 0.351	0.528	− 0.139	− 0.599; 0.320	0.553	− 0.464	− 2.819; 1.891	0.700	− 0.170	− 0.861; 0.521	0.630	− 0.180	− 1.771; 1.411	0.825
Physical anhedonia	Smoking initiation	58	− 0.001	− 0.006; 0.004	0.678	− 0.000	− 0.005; 0.004	0.850	0.003	− 0.009; 0.015	0.626	0.002	− 0.005; 0.009	0.530	0.010	− 0.006; 0.027	0.222
Smoking initiation	Social anhedonia	98	− 0.454	− 0.935; 0.028	0.065	− 0.414	− 0.870; 0.043	0.076	− 0.579	− 2.770; 1.611	0.605	− 0.469	− 1.131; 0.194	0.166	− 0.326	− 1.963; 1.311	0.697
Social anhedonia	Smoking initiation	57	− 0.001	− 0.005; 0.004	0.746	− 0.001	− 0.005; 0.004	0.754	0.005	− 0.004; 0.014	0.294	0.002	− 0.004; 0.008	0.578	0.004	− 0.008; 0.015	0.523
**Smoking and adolescent PENS**																	
Smoking initiation	Paranoia/hallucinations	57	0.338	− 0.076; 0.753	0.110	0.282	− 0.135; 0.700	0.185	0.437	− 1.517; 2.391	0.663	0.073	− 0.518; 0.665	0.808	− 0.056	− 1.188; 1.077	0.923
Paranoia/hallucinations	Smoking initiation	24	− 0.001	− 0.013; 0.011	0.880	− 0.002	− 0.013; 0.009	0.720	− 0.015	− 0.045; 0.015	0.330	− 0.006	− 0.022; 0.010	0.461	− 0.015	− 0.043; 0.014	0.331
Smoking initiation	Cognitive disorganisation	57	1.077	0.576; 1.578	**2.55 × 10**^**–5**^	1.030	0.518; 1.541	**8.02 × 10**^**–5**^	2.321	− 0.013; 4.654	0.056	1.267	0.566; 1.969	**6.31 × 10**^**–4**^	1.352	− 0.101; 2.805	0.074
Cognitive disorganisation	Smoking initiation	28	0.006	− 0.003; 0.015	0.190	0.004	− 0.004; 0.012	0.307	− 0.022	− 0.043; − 0.001	0.059	0.002	− 0.009; 0.013	0.720	− 0.001	− 0.020; 0.019	0.945
Smoking initiation	Anhedonia	57	0.061	− 0.444; 0.568	0.811	0.058	− 0.420; 0.536	0.812	− 0.841	− 3.209; 1.528	0.490	− 0.075	− 0.754; 0.603	0.828	− 0.218	− 1.526; 1.090	0.745
Anhedonia	Smoking initiation	30	− 0.003	− 0.011; 0.006	0.533	− 0.002	− 0.010; 0.006	0.599	− 0.000	− 0.018; 0.018	0.965	− 0.000	− 0.012; 0.011	0.944	0.002	− 0.019; 0.023	0.864
Smoking initiation	Negative symptoms	57	0.523	0.074; 0.973	**0.023**	0.506	0.105; 0.906	**0.013**	1.221	− 0.914; 3.356	0.267	0.450	− 0.125; 1.025	0.125	0.529	− 0.492; 1.550	0.315
Negative symptoms	Smoking initiation	25	− 0.002	− 0.014; 0.009	0.712	− 0.003	− 0.014; 0.008	0.609	− 0.012	− 0.042; 0.018	0.436	− 0.004	− 0.019; 0.012	0.656	0.004	− 0.023; 0.031	0.770

GSMR, Generalized Summary-based Mendelian Randomization; MR-IVW, inverse-variance weighted Mendelian randomization; n SNPs, number of variants remaining in analyses after those identified as Heidi-outliers or with residual LD at r^2^ > .1 were removed; PENS, psychotic experiences and negative symptom traits; SNPs identified as having residual LD and as Heidi outliers were also excluded from MR Egger, Weighted Median and Weighted Mode analyses; Bold text indicates significant *p* values at *p* < 0.05.

^a^Summary statistics for smoking initiation excluded UK Biobank participants in analyses on major depression and adult psychotic experiences to avoid overlapping samples (N = 249,171).

Evidence of a causal effect of smoking initiation on liability to major depression was found, and replicated in Weighted Median MR. However, this effect was not replicated in Weighted Mode MR and the GSMR effect size fell outside of the MR-Egger confidence intervals. Therefore, biased results due to violation of the instrumental variable assumptions cannot be ruled out. The MR-Egger intercept was significantly different from zero (*p* = 0.048), suggesting that results may have been affected by directional pleiotropy. No consistent evidence of a causal effect of depression liability on smoking initiation was detected.

Evidence of a causal effect of smoking initiation on liability for bipolar disorder was reported in GSMR and Weighted Median analyses with a similar effect size observed in MR-Egger, but not replicated in the Weighted Mode analysis. No evidence of reverse causation was found.

Evidence of a causal effect of smoking initiation on cognitive disorganisation was observed in GSMR and Weighted Median analyses. Effect sizes fell within the MR-Egger confidence intervals but was not replicated in Weighted Mode MR. We found a causal effect of smoking initiation on negative symptoms in the main MR analysis, but this effect was not replicated in MR sensitivity analyses. No evidence of reverse causation was observed.

## Discussion

This study aimed to investigate systematically the degree of overlapping common genetic influences between smoking behaviours and psychiatric traits and disorders across adolescence and adulthood. We found evidence of overlapping genetic influences at a genome-wide level between smoking behaviours and psychiatric disorders and with some psychotic experiences and negative symptom traits reported by adolescents and adults in the community. For schizophrenia and depression, the overlap in common genetic influences shown with smoking frequency and current smoking status remained after controlling for genetic influences on known covariates, namely cannabis use, alcohol use, risk taking and insomnia. Genetic associations between smoking behaviours and most positive psychotic experiences were explained by genetic influences shared with the covariates. We found evidence of causal effects of smoking initiation on adolescent cognitive and (to some degree) negative symptoms as well as on schizophrenia, depression and bipolar disorder. These findings hint at plausible pathways by which smoking during adolescence could be involved in the development of psychiatric disorders. However, evidence of causation should be considered moderate to weak because not all sensitivity analyses confirmed findings.

Our GSEM findings support the hypothesis that schizophrenia and depression share genetic pathways with smoking frequency and persistent smoking. This shared genetic aetiology may point toward common biological pathways such as those involving nicotine, the principal pharmacologically active component of smoking that acts as an agonist on the nicotinic acetylcholine receptor (nAChR). Variants within the CHRNA5‐A3‐B4 gene cluster which encodes for subunits of nAChR are among the most robust associations with nicotine dependence^50–53^ and have also been implicated in schizophrenia^18^. Presynaptic activation of nAChR stimulates the release of several neurotransmitters including dopamine, serotonin and glutamate^54–57^. Dysregulation of dopaminergic and glutamatergic pathways could both explain why some people may be more susceptible to the positive reinforcing effects of smoking^58^ and have an increased vulnerability to develop schizophrenia^59^.

We found support for a causal role of smoking initiation on liability to develop schizophrenia, depression and bipolar disorder, corroborating recent findings^33,34^ whilst applying different Mendelian randomization methods. Compared to previous MR studies, we employed a method to remove likely pleiotropic variants from genetic instruments which aims to reduce confounding from biological pleiotropy. Despite this, not all sensitivity analyses yielded consistent findings in our study, and therefore biased causal effect estimates due to pleiotropy or a proportion of invalid instruments cannot be ruled out. Interestingly, our GSEM findings indicated that the covariates accounted for genetic overlap between schizophrenia and smoking initiation, raising the possibility that these traits may be involved in causal pathways of smoking initiation on schizophrenia or that the Mendelian randomization methods may not have adequately controlled for pleiotropy. Our results of a possible causal effect of smoking initiation on schizophrenia and depression (and a small causal effect of schizophrenia on smoking initiation) concurs with meta-analytic findings of longitudinal studies^21,23^. The action of nicotine on nAChR could underlie a mechanism by which smoking causally influences mental health. Chronic exposure to nicotine may result in long-lasting alterations of dopaminergic and cholinergic pathways, leading to an increase in risk of psychiatric disorders^60–62^. Beyond nicotine, other toxic compounds released during combustion of tobacco cause neuro-inflammation and oxidative stress^63^, factors that are associated with psychiatric disorders^64–66^.

To our knowledge, this is the first study to report that genome-wide genetic influences on bipolar disorder overlap with those on smoking frequency and initiation. This finding supports recent evidence of an association between polygenic scores for bipolar disorder and nicotine dependence^17^. Genetic overlap between bipolar disorder and smoking quantity was accounted for by genetic influences on the covariates. Interestingly, we found that genetic influences on smoking initiation was negatively associated with genetic influences on bipolar disorder after accounting for genetic overlap with the covariates, whereas the bivariate genetic correlation between these two phenotypes was positive. This negative genetic association was likely masked by overlapping genetic influences of the covariates. As such, currently identified common genetic variation shared between smoking and bipolar disorder is unlikely to explain causal pathways or may reflect complex pathways underlying multiple, related addiction, personality and psychiatric phenotypes.

Until recently, research into the genetic aetiology underlying the association between psychotic experiences in the community and smoking behaviours was lacking. Here, we report novel evidence that smoking behaviours share genetic influences with some types of psychotic experiences during adulthood (notably with visual and auditory hallucinations) and with hypomania. Interestingly, unlike our findings for schizophrenia and depression, the genetic overlap between most psychotic experiences and smoking was shared with the covariates. This suggest that overlapping genome-wide genetic influences (captured by current GWASs) that act on both psychotic experiences and smoking behaviours are likely shared with co-occurring traits such as other substance use, sleep problems and risk taking, or mechanisms underlying these traits.

Our findings suggest that smoking may increase a propensity to experience cognitive and negative psychotic experiences during adolescence, although the association with negative symptom traits was not confirmed by sensitivity analyses. Our findings that smoking may cause early cognitive and negative symptoms during adolescence could hint at developmental pathways by which smoking could increase the risk of developing psychiatric disorders. A recent preclinical study found that during adolescence, exposure to nicotine could lead to persistent alteration of neurotransmitter pathways^61^ likely relevant to psychotic experiences. Smoking initiation and positive psychotic experiences in adulthood did not appear to be causally related. This is somewhat surprising given the known phenotypic association between psychotic experiences and psychiatric disorders^67–72^ and could be explored further. Evidence suggests that psychotic experiences in adults have somewhat different underlying causal influences compared to psychotic experiences during adolescence^73^ which may explain that lack of causal associations between smoking and psychotic experiences in later life. We also note that the GWASs on positive psychotic experiences were based on a small number of cases, which could have resulted in low power to detect causal effects in our study.

This study had limitations to be considered. In some genomic multiple regression models, the estimated residual variances were imprecise due to small GWAS sample sizes, particularly for schizotypy and adolescent PENS. To mitigate this, we performed GSEM models only for traits that had significant bivariate genetic correlations. However, non-significant genetic correlations may have reflected smaller GWAS sample sizes rather than the absence of genetic overlap, and these analyses should be revisited once more powerful summary statistics become available. It is also possible that genetic influences on co-occurring factors other than those we considered (such as exposure to adverse life events or sociodemographic characteristics) could account for the genetic overlap between smoking and psychiatric disorders. Our Mendelian randomization analyses that used instruments from psychotic experiences summary statistics requires replication once better-powered GWASs for these psychiatric traits become available. We performed Mendelian randomization using instruments for smoking initiation, which has the advantage of being a trait measurable to the whole adolescent and adult population (unlike number of cigarettes per day, which only applies to smokers). Nevertheless, it is noted that smoking initiation may partly pick up on traits such as risk taking or impulsivity. Future studies should consider using instruments for objective measures of smoking on samples stratified by smoking status to confirm our results. However, such designs have their own limitations since gene-environment interactions are likely (genes affecting tobacco consumption will only do so in those who took up smoking) and in such cases Mendelian randomization on stratified samples may introduce collider bias^74^. Possible pleiotropic effects can be further investigated using multivariable Mendelian randomization, but GWASs with overlapping samples for exposures and confounders (as was the case here) require additional adjustments and individual-level phenotypic data. The GWAS on smoking initiation included a small number of ALSPAC participants and overlapping samples may have inflated Mendelian randomization estimates in analyses with adolescent PENS. Despite our approach to exclude possible pleiotropic instruments from Mendelian randomization analyses, the sensitivity analyses indicated that we cannot rule out bias due to violation of the instrumental variable assumptions. Finally, results based on GWASs from large cohort and biobank studies may be affected by ascertainment bias.

In conclusion, there is genetic overlap between smoking behaviours and schizophrenia and depression, and we show it exists beyond genetic influences shared with other risk factors. Furthermore, smoking appears to play a role in the development of traits relating to mental illness during adolescence and our findings strengthen the evidence base for smoking as a causal risk associated with psychiatric disorders. Our study indicates that the mental health risks of smoking during adolescence require further investigation. Exploration of the biological links underlying smoking and psychiatric illness, and smoking interventions as part of mental health prevention strategies, are well-justified based on current evidence.

## Methods

### Samples and measures

Summary statistics for participants of European descent used in the analyses are presented in Table 3. Smoking behaviours^50^ included smoking initiation, a binary phenotype with smokers defined as those who reported having ever smoked regularly. The average number of cigarettes per day was assessed in current and former smokers, with never-smokers excluded. Age of smoking initiation was defined as the age at which current or former smokers started smoking regularly. Current smoking was assessed among smokers and coded as either current smokers or former smokers.

**Table 3:** Summary statistics, phenotypes and sample sizes.

Phenotypes	GWAS N	SNP-h^2^ (SE)	Samples
**Smoking behaviour**^50^			GSCAN
Smoking initiation	632,802	0.0885 (0.0029)	
Cigarettes per day	263,954	0.0625 (0.0068)	
Age of smoking initiation	262,990	0.0423 (0.0022)	
Current smoking ^a^	312,821	0.0490 (0.0028)	
**Adolescent PENS**^75^			Meta-GWAS of TEDS^75^ (mean age 16.32 years), ALSPAC^76,77^ (mean age 16.76 years) and CATSS^78^ (mean age 18.31 years)
Paranoia and hallucinations	8665	− 0.0042 (0.0352)	
Cognitive disorganization	6297	0.1048 (0.0566)	
Anhedonia	6579	0.0797 (0.0479)	
Negative symptoms	10,098	− 0.0222 (0.0316)	
**Schizotypy**^81^			Northern Finland Birth Cohort 1996 (NFBC; at age 31 years)^79^
Hypomania	3967	0.3732 (0.1011)	
Perceptual aberrations	4057	0.3037 (0.0916)	
Physical anhedonia	3988	0.3655 (0.0965)	
Social anhedonia	4025	0.2950 (0.0826)	
**Positive PE**			UK Biobank (aged 40 – 69 years) obtained from the Neale Lab (http://www.nealelab.is/uk-biobank)
Auditory hallucinations	117,503	0.0709 (0.0255)	
Visual hallucinations	116,787	0.1032 (0.0224)	
Delusions of persecution	117,794	0.0910 (0.0521)	
Delusions of reference	117,731	0.0666 (0.0499)	
**Psychiatric disorders**			PGC (https://www.med.unc.edu/pgc/results-and-downloads)
Schizophrenia^85^	105,318	0.2666 (0.0067)	
Bipolar disorder^86^	41,653	0.2999 (0.0102)	
Major Depression^16^	173,005	0.0999 (0.0042)	
**Covariates**			
Lifetime cannabis use^32^	162,082	0.1880 (0.0082)	ICC and UK Biobank
Alcohol consumption^50^	537,349	0.0396 (0.0017)	GSCAN
Risk taking (Neale Lab)	348,549	0.0560 (0.0018)	UK Biobank
Insomnia^27^	113,006	0.0863 (0.0061)	UK Biobank

*GSCAN* GWAS and Sequencing Consortium of Alcohol and Nicotine use, *PENS* psychotic experiences (PE) and negative symptom traits, *TEDS* Twins Early Development Study, *ALSPAC* Avon Longitudinal Study of Parents and Children, *CATSS* Child and Adolescent Twin Study in Sweden, *PGC* Psychiatric Genomics Consortium, *ICC* International Cannabis Consortium.

Summary statistics for adolescent PENS were obtained from a mega-GWAS of four continuous scales of PENS, assessed when participants were 15 to 19 years old. Ethical approval for the original adolescent PENS GWAS^75^ was obtained for TEDS^76^ from the Institute of Psychiatry ethics committee (ref: 05/Q0706/228), for ALSPAC^77,78^ from the ALSPAC Ethics and Law Committee and the Local Research Ethics Committees, and for CATSS^79^ from the Karolinska Institute Ethical Review Board. All research participants and their parents granted informed consent.

Summary statistics for schizotypy in adulthood^80,81^ were available for four continuous schizotypy scales^82–84^. Positive psychotic experiences in adults were assessed using four dichotomous items on lifetime occurrence. The average age of onset of psychotic experiences was 31.6 (s.d. = 17.6) years. Psychiatric disorders were defined based on standard diagnostic criteria and assessed during clinical interviews or obtained from health records and self-report^16,85,86^.

For covariates in genomic multiple regression, we selected phenotypes considered to be likely confounders of the association between smoking and psychotic experiences/psychiatric disorders^9–11,19–23^ and for which GWAS summary statistics were available. Summary statistics were obtained for: cannabis use^32^, a binary phenotype for whether participants had ever used cannabis; alcohol consumption^50^, defined as average number of weekly drinks; risk taking was assessed with the item “*Would you describe yourself as someone who takes risks?*”; and insomnia defined as having trouble falling asleep at night or waking up in the middle of the night^27^.

Additional details on summary statistics are provided in Supplementary Table S1 and the Supplementary Methods. The Birkbeck Department of Psychological Sciences’ Ethics Committee approved this study and all research was performed in accordance with institutional guidelines.

### Analyses

Prior to analyses, single nucleotide polymorphisms (SNPs) with incomplete association statistics and strand ambiguous and non-biallelic SNPs were excluded. Variants were matched and allele orders harmonized to the 1000 Genomes (phase 3) reference panel for European ancestry and restricted to HapMap 3 variants. Variants were excluded based on INFO scores < 0.9 and minor allele frequency (MAF) < 0.01. INFO scores were not provided in the summary statistics for smoking behaviours and drinks per week and were filtered on INFO < 0.3 by the study authors^50^.

#### LD score regression

Genetic correlations (r_g_) were estimated using linkage disequilibrium (LD) score regression^87,88^ on a liability scale based on population prevalences of 1% for schizophrenia, 15% for major depression and 2% for bipolar disorder^89–91^. Covariance intercepts were left unconstrained in analyses with overlapping samples (GWASs for smoking behaviours, depression and psychotic experiences in adults included UK Biobank participants; GWASs for smoking behaviours and adolescent PENS included ALSPAC participants). Correction for multiple testing (60 tests) was performed using Benjamini–Hochberg correction at a false discovery rate (FDR) of 0.05.

#### Genomic structural equation modelling

To investigate genetic overlap between psychiatric phenotypes and smoking behaviours after accounting for the genetic influences on confounds, Genomic Structural Equation Modelling (Genomic SEM)^44^ was used to model shared genetic architecture using genetic covariance structures.

Summary statistics were converted to include LD scores in LD score regression software using the parameters described above. Genomic covariance structures were computed, and genomic multiple regression models specified in the Genomic SEM package for R version 3.5.2^92^ for phenotype pairs that had nominally significant genetic correlations (at *p* < 0.05). Models allowed for genetic covariation between smoking phenotypes and the covariates included as predictors and regressed onto psychiatric outcomes. Standardized estimates were reported thereby allowing the association between a given predictor and each outcome to be interpreted as the genetic correlation conditional on all other predictors. Conditional genetic correlations were considered statistically significant at *p* < 0.05.

#### Mendelian randomization

Mendelian randomization (MR)^93^ was performed to test for bi-directional causal associations between smoking initiation and psychiatric phenotypes using summary statistics (see also Supplementary Methods).

Generalised Summary-data-based Mendelian Randomisation (GSMR) was used as the main method due to its advantages of accounting for sampling variation in the exposure and outcome GWASs and for residual LD structure between variants used as instrumental variables (IVs)^45^. As a baseline to compare against sensitivity analyses, we also report MR-IVW^46^ results. MR-Egger regression, Weighted Median and Weighted Mode MR were conducted as sensitivity analyses as these methods make different assumptions to GSMR and MR-IVW by allowing for a proportion of invalid IVs in the analyses^47–49^. To assess whether MR-Egger will be an appropriate sensitivity analysis, we computed the I^2^ statistic, with I^2^ > 0.9 indicating that MR-Egger results can be considered reliable^94^. MR-Egger is typically less powerful to detect significant effects, but the MR-Egger confidence intervals are expected overlap with valid effect sizes from other methods. Directional pleiotropy was assessed using the MR-Egger intercept test. An intercept significantly different from zero indicates that MR-Egger causal estimates may be more robust compared to GSMR estimates. The strength of association between the IVs and exposures was assessed using the mean F-statistic, with F > 10 considered adequate instrument strength. Consistent findings across MR methods indicates that results from GSMR are less likely to be biased due to violations of IV assumptions.

Summary statistics for major depression excluded 23andMe participants. To ensure at least 20 IVs were used, genome-wide significant variants were however obtained from the publication that did include the 23andMe participants^16^. Major depression and adult psychotic experiences summary statistics included UK Biobank participants. To avoid overlapping samples in these MR analyses, we used a version of the smoking initiation summary statistics that excluded UK Biobank participants (N = 249,171) and selected IVs at a *p* value threshold of 5 × 10^–7^, resulting in 20 independent depression variants to use as IVs in these MR analyses. Only seven variants reached genome-wide significance in the summary statistics for bipolar disorder^86^. IVs for bipolar disorder were obtained from the publication of a recent meta-GWAS, also conducted on Psychiatric Genomics Consortium samples, but for which full summary statistics were not available^95^. For all other exposures, IVs were identified using the clumping algorithm in PLINK^96^ based on an r^2^ threshold = 0.05 within a 500 kb window. Recent GWASs on psychotic experiences have not yet been replicated using equivalent measures in independent samples or have been based on relatively small sample sizes. Therefore, IVs for psychotic experiences were selected at *p* < 5 × 10^–5^.

Analyses were performed in GSMR and MR Base R packages^45,97^. GCTA^98^ was used to calculate the LD structure between lead variants based on the 1000 Genomes (phase 3) reference panel for European ancestry. IVs excluded from GSMR analyses due to being Heidi-outliers and in residual LD at r^2^ > 0.1 were also removed from MR sensitivity analyses. MR was conducted for smoking initiation but not for smoking phenotypes assessed in smokers only. This is because variant associations among smokers may not explain smoking liability in samples that include non-smokers. Smoking initiation is also temporally relevant to the adolescent PENS since most smokers initiate smoking during adolescence^99^. Significance thresholds were set at *p* < 0.05.

## Data and code availability

GWAS summary statistics used in the analyses for this paper are available at https://atlas.ctglab.nl/ and at https://www.med.unc.edu/pgc/download-results/. Summary data for adolescent psychotic experiences and for schizotypy were obtained from the authors with permission from the participating cohorts. Code to perform genomic structural equation modelling of GWAS summary statistics within Genomic SEM can be found at https://github.com/MichelNivard/GenomicSEM/wiki. Code to perform the Mendelian randomization analyses is available at https://cnsgenomics.com/software/gsmr/ and at https://www.mrbase.org/.

## Supplementary Information

Supplementary Information.
