# Supplementary material for: Genetic overlap and causal associations between smoking behaviours and mental health
Source: Sci Rep. 2021 Jul 21;11:14871. doi: 10.1038/s41598-021-93962-7 (PMC8295327; doi:10.1038/s41598-021-93962-7)
Supplement: Supplementary file 1 — Supplementary Information. [file 41598_2021_93962_MOESM1_ESM.pdf]

Supplementary Material: Genetic overlap and causal associations between smoking behaviours and mental health

Wikus Barkhuizen[1,2], Frank Dudbridge[3] and Angelica Ronald[1].

1) Centre for Brain and Cognitive Development, Department of Psychological Sciences, Birkbeck, University of London, UK; 2) Department of Clinical, Educational and Health Psychology, University College London, London, UK; 3) Department of Health Sciences, University of Leicester, UK.

Table of Content

Supplementary Table S1. Genome wide association study sample sizes and SNP-heritability estimates 2
Supplementary Table S2. MR-Egger tests for heterogeneity and directional horizontal pleiotropy 3
Supplementary Table S3. Number of variants excluded due to residual LD and during Heidi-outlier analyses, and assessment of instrument strength…………………………...…………………………………………….4
Supplementary Table S4. Odds ratios from Mendelian randomization analyses on binary outcomes……………5
Supplementary Figure S1. Genetic correlations between smoking behaviours, psychotic experiences and psychiatric disorders with covariates used in genomic multiple regression.. 6
Supplementary Figure S2. Genomic multiple regression models for cigarettes per day as a predictor of psychiatric disorders and psychotic experiences... 7
Supplementary Figure S3. Genomic multiple regression models for smoking initiation as a predictor of psychiatric disorders and psychotic experiences... 9
Supplementary Figure S4. Genomic multiple regression models for age of smoking initiation as a predictor of psychiatric disorders and psychotic experiences... 11
Supplementary Figure S5. Genomic multiple regression models for current smoking status as a predictor of psychiatric disorders and psychotic experiences... 13
Supplementary Figure S6. Generalised Summary-Based Mendelian Randomization analyses between psychiatric disorders and smoking initiation.. 14
Supplementary Figure S7. Generalised Summary-Based Mendelian Randomization between positive psychotic experiences in adulthood and smoking initiation 15
Supplementary Figure S8. Generalised Summary-Based Mendelian Randomization between schizotypy in adulthood and smoking initiation.. 16
Supplementary Figure S9. Generalised Summary-Based Mendelian Randomization between adolescent psychotic experiences and negative symptom traits and smoking initiation 17
Supplementary Figure S10. MR-Egger, Weighted Median and Weighted Mode Mendelian randomization sensitivity analyses between psychiatric disorders and smoking initiation.. 18
Supplementary Figure S11. MR-Egger, Weighted Median and Weighted Mode Mendelian randomization sensitivity analyses between positive psychotic experiences in adulthood and smoking initiation 19
Supplementary Figure S12. MR-Egger, Weighted Median and Weighted Mode Mendelian randomization sensitivity analyses between schizotypy in adulthood and smoking initiation 20
Supplementary Figure S13. MR-Egger, Weighted Median and Weighted Mode Mendelian randomization sensitivity analyses between adolescent psychotic experiences and negative symptom traits and smoking initiation 21
Supplementary Methods..……………………………………………………………………………..22

Supplementary Table S1. Genome-wide association study sample sizes and SNP-heritability estimates

	GWAS N	N cases	QC-positive SNPs	SNP-h^2	SE	*p*
**Tobacco use**[1]						
Smoking initiation	632,802	311,629	6,314,516	0.0885	0.0029	<.001
Cigarettes per day	263,954	*Continuous*	6,390,544	0.0625	0.0068	<.001
Age of smoking initiation	262,990	*Continuous*	6,395,535	0.0423	0.0022	<.001
Current smoking [a]	312,821	92,573	6,424,129	0.049	0.0028	<.001
**Adolescent psychotic experiences and negative symptom traits**[2]						
Paranoia and hallucinations	8,665	*Continuous*	3,363,829	-0.0042	0.0352	.453
Cognitive disorganization	6,297	*Continuous*	3,363,829	0.1048	0.0566	.032
Anhedonia	6,579	*Continuous*	3,363,829	0.0797	0.0479	.048
Negative symptoms	10,098	*Continuous*	3,363,829	-0.0222	0.0316	.241
**Schizotypy during adulthood**[3]						
Hypomania	3,967	*Continuous*	5,493,986	0.3732	0.1011	<.001
Perceptual aberrations	4,057	*Continuous*	5,493,986	0.3037	0.0916	<.001
Physical anhedonia	3,988	*Continuous*	5,493,986	0.3655	0.0965	<.001
Social anhedonia	4,025	*Continuous*	5,493,986	0.2950	0.0826	<.001
**Positive psychotic experiences**						
Auditory hallucinations	117,503	2,009	6,443,634	0.0709	0.0255	.003
Visual hallucinations	116,787	3,768	6,443,706	0.1032	0.0224	<.001
Delusions of persecution	117,794	932	6,443,695	0.0910	0.0521	.040
Delusions of reference	117,731	822	6,443,693	0.0666	0.0499	.091
**Psychiatric disorders**						
Schizophrenia[4]	105,318	40,675	4,593,674	0.2666	0.0067	<.001
Bipolar disorder[5]	41,653	20,129	5,083,505	0.2999	0.0102	<.001
Major Depression[6]	173,005	59,851	5,488,968	0.0999	0.0042	<.001

SNP-h^2 = univariate SNP heritability; [a] Current smoking cases are current smokers (compared to ex-smokers); SNP heritabilities converted to liability scale for LD score regression using binary phenotypes; Effective sample size used for adolescent PENS in LD score regression analyses (paranoia and hallucinations = 7970.416; cognitive disorganization = 5082.760; anhedonia = 6068.311; parent-rated negative symptoms = 8763.295).

Supplementary Table S2. MR-Egger tests for heterogeneity and directional horizontal pleiotropy

Exposure		Outcome	MR-Egger intercept test			Cochran's Q statistic		
			Intercept	SE	*p*	Q	Q_df	p
Smoking initiation	→	Schizophrenia	0.004	0.010	0.695	285.73	90	3.19E-22
Schizophrenia	→	Smoking initiation	-0.002	0.001	0.043	275.30	123	1.12E-13
Smoking initiation	→	Major depression	0.046	0.021	0.048	50.19	18	$7.07x10^{-5}$
Major depression	→	Smoking initiation	-0.136	0.045	0.006	43.10	27	0.026
Smoking initiation	→	Bipolar disorder	0.001	0.011	0.910	187.07	104	1.09E-06
Bipolar disorder	→	Smoking initiation	0.008	0.004	0.084	33.93	16	0.006
Smoking initiation	→	Auditory hallucinations	0.001	0.001	0.583	38.86	19	0.008
Auditory hallucinations	→	Smoking initiation	-0.003	0.002	0.170	97.38	88	0.232
Smoking initiation	→	Visual hallucinations	0.001	0.001	0.399	18.77	19	0.472
Visual hallucinations	→	Smoking initiation	-0.002	0.002	0.541	91.25	79	0.163
Smoking initiation	→	Delusions of reference	-0.000	0.000	0.665	20.42	19	0.369
Delusions of reference	→	Smoking initiation	0.001	0.002	0.502	101.81	103	0.515
Smoking initiation	→	Delusions of persecution	0.000	0.001	0.636	18.95	19	0.460
Delusions of persecution	→	Smoking initiation	0.001	0.002	0.798	114.13	92	0.059
Smoking initiation	→	Hypomania	0.002	0.013	0.875	111.16	96	0.138
Hypomania	→	Smoking initiation	0.000	0.001	0.593	85.57	66	0.053
Smoking initiation	→	Perceptual aberrations	-0.006	0.013	0.645	123.92	96	0.029
Perceptual aberrations	→	Smoking initiation	0.000	0.001	0.906	54.33	52	0.386
Smoking initiation	→	Physical anhedonia	0.003	0.014	0.800	129.08	96	0.014
Physical anhedonia	→	Smoking initiation	-0.001	0.001	0.461	72.86	56	0.064
Smoking initiation	→	Social anhedonia	0.001	0.013	0.908	112.81	96	0.116
Social anhedonia	→	Smoking initiation	-0.001	0.001	0.167	58.82	55	0.337
Smoking initiation	→	Paranoia/hallucinations	-0.001	0.011	0.919	57.29	55	0.390
Paranoia/hallucinations	→	Smoking initiation	0.002	0.001	0.315	29.96	22	0.119
Smoking initiation	→	Cognitive disorganisation	-0.015	0.014	0.290	44.48	55	0.844
Cognitive disorganisation	→	Smoking initiation	0.004	0.001	0.011	30.04	26	0.266
Smoking initiation	→	Anhedonia	0.011	0.014	0.448	64.98	55	0.168
Anhedonia	→	Smoking initiation	0.000	0.001	0.793	30.33	28	0.348
Smoking initiation	→	Negative symptoms	-0.008	0.012	0.515	73.68	55	0.047
Negative symptoms	→	Smoking initiation	0.001	0.001	0.487	26.71	23	0.269

LD SNPs, SNPs with residual LD at r2 > 0.1 removed from analysis; n SNPs, number of genetic variants used as instruments in Mendelian randomization analyses.

Supplementary Table S3. Number of variants excluded due to residual LD and during Heidi-outlier analyses, and assessment of instrument strength

Exposure	Outcome	Heidi SNPs	LD SNPs	n SNP	Mean F-statistic	I^2
Smoking initiation	Schizophrenia	10	3	92	31.67	0.97
Schizophrenia	Smoking initiation	12	11	125	44.64	0.98
Smoking initiation	Major depression	0	0	20	31.72	0.97
Major depression	Smoking initiation	3	0	29	35709.38	0.97
Smoking initiation	Bipolar disorder	4	3	106	31.11	0.97
Bipolar disorder	Smoking initiation	0	0	18	34.45	0.97
Smoking initiation	Auditory hallucinations	0	0	21	31.68	0.97
Auditory hallucinations	Smoking initiation	0	0	90	18.69	0.93
Smoking initiation	Visual hallucinations	0	0	21	31.68	0.97
Visual hallucinations	Smoking initiation	0	0	81	18.66	0.94
Smoking initiation	Delusions of reference	0	0	21	31.68	0.97
Delusions of reference	Smoking initiation	0	0	105	18.85	0.93
Smoking initiation	Delusions of persecution	0	0	21	31.68	0.97
Delusions of persecution	Smoking initiation	0	0	94	18.39	0.93
Smoking initiation	Hypomania	0	3	98	32.22	0.97
Hypomania	Smoking initiation	0	0	68	18.76	0.95
Smoking initiation	Perceptual aberrations	0	3	98	32.22	0.97
Perceptual aberrations	Smoking initiation	0	0	54	18.87	0.95
Smoking initiation	Physical anhedonia	0	3	98	32.22	0.97
Physical anhedonia	Smoking initiation	1	0	58	18.61	0.95
Smoking initiation	Social anhedonia	0	3	98	32.22	0.97
Social anhedonia	Smoking initiation	1	0	57	18.34	0.94
Smoking initiation	Paranoia/hallucinations	0	1	57	32.06	0.97
Paranoia/hallucinations	Smoking initiation	0	0	24	17.55	0.94
Smoking initiation	Cognitive disorganisation	0	1	57	32.06	0.97
Cognitive disorganisation	Smoking initiation	0	0	28	18.67	0.95
Smoking initiation	Anhedonia	0	1	57	32.06	0.97
Anhedonia	Smoking initiation	0	0	30	19.06	0.95
Smoking initiation	Negative symptoms	0	1	57	32.06	0.97
Negative symptoms	Smoking initiation	0	0	25	18.17	0.95

Supplementary Table S4 – Odds ratios from Mendelian randomization analyses on binary outcomes

**Exposure**	**Outcome**	**MR-IVW results**			**GSMR results**			**MR-Egger**			**Weighted Median**			**Weighted Mode**		
		OR	**95% *CI***	***p***	***OR***	**95% *CI***	***p***	***OR***	**95% *CI***	***p***	***OR***	**95% *CI***	***p***	***OR***	**95% *CI***	***p***
Smoking initiation	Schizophrenia	1.94	1.37; 2.76	**<.001**	1.79	1.45; 2.20	**<.001**	1.36	0.22; 8.45	0.744	1.47	1.03; 2.10	**0.033**	1.07	0.40; 3.08	0.901
Schizophrenia	Smoking initiation	1.02	1.01; 1.02	**<.001**	1.02	1.01; 1.02	**<.001**	1.05	1.02; 1.08	**0.003**	1.02	1.01; 1.03	**<.001**	1.03	1.00; 1.07	**0.036**
Smoking initiation[a]	Major depression	1.54	1.27; 1.88	**<.001**	1.44	1.28; 1.62	**<.001**	0.43	0.13; 1.42	0.183	1.38	1.13;1.68	**0.001**	1.06	0.72; 1.56	0.771
Major depression	Smoking initiation	1.00	1.00; 1.00	0.930	1.00	1.00; 1.00	0.638	1.14	1.05; 1.25	**0.006**	1.00	1.00; 1.00	0.642	1.00	0.99; 1.00	0.449
Smoking initiation	Bipolar disorder	2.46	1.68; 3.61	**<.001**	2.25	1.67; 3.03	**<.001**	2.21	0.34; 14.38	0.407	2.23	1.41; 3.52	**0.001**	2.44	0.79; 7.56	0.124
Bipolar disorder	Smoking initiation	1.01	-0.01; 0.03	0.415	1.01	0.99; 1.02	0.356	0.91	0.81; 1.02	0.112	1.00	0.98; 1.02	0.767	0.99	0.96; 1.02	0.578
Smoking initiation	Auditory hallucinations	1.00	0.99; 1.01	0.447	1.00	1.00; 1.01	0.323	0.99	0.93; 1.05	0.680	1.00	0.99; 1.01	0.813	1.00	0.98; 1.02	0.709
Auditory hallucinations	Smoking initiation	0.89	0.52; 1.52	0.675	0.89	0.53; 1.49	0.651	1.86	0.58; 6.01	0.299	0.78	0.37; 1.67	0.529	0.65	0.09; 4.47	0.662
Smoking initiation	Visual hallucinations	1.00	0.99; 1.01	0.859	1.00	0.99; 1.01	0.820	0.98	0.92; 1.03	0.423	1.00	0.98; 1.01	0.805	1.00	0.97; 1.03	0.798
Visual hallucinations	Smoking initiation	1.24	0.81; 1.90	0.314	1.22	0.81; 1.83	0.345	1.65	0.61; 4.46	0.329	1.21	0.68; 2.16	0.517	1.19	0.30; 4.66	0.802
Smoking initiation	Delusions of reference	1.00	1.00; 1.01	0.276	1.00	1.00; 1.01	0.280	1.01	0.98; 1.04	0.543	1.00	0.99; 1.01	0.669	1.00	0.98; 1.01	0.545
Delusions of reference	Smoking initiation	1.00	0.48; 2.08	0.994	0.96	0.45; 2.05	0.914	0.64	0.14; 2.87	0.560	0.79	0.27; 2.34	0.672	1.48	0.08; 28.46	0.795
Smoking initiation	Delusions of persecution	1.00	1.00; 1.01	0.168	1.00	1.00; 1.01	0.193	1.00	0.97; 1.02	0.820	1.00	1.00; 1.01	0.441	1.00	0.99; 1.01	0.807
Delusions of persecution	Smoking initiation	1.17	0.52; 2.62	0.710	1.16	0.54; 2.48	0.700	0.96	0.17; 5.31	0.960	0.72	0.25; 2.09	0.549	0.36	0.02; 5.28	0.454
Hypomania	Smoking initiation	1.00	0.99; 1.00	0.644	1.00	0.99; 1.00	0.565	1.00	0.99; 1.01	0.494	1.00	0.99; 1.00	0.455	1.00	0.98; 1.01	0.609
Perceptual aberrations	Smoking initiation	1.00	0.99; 1.00	0.731	1.00	0.99; 1.00	0.867	1.00	0.99; 1.01	0.792	1.00	0.99; 1.01	0.729	1.00	0.99; 1.01	0.951
Physical anhedonia	Smoking initiation	1.00	0.99; 1.00	0.678	1.00	0.99; 1.00	0.850	1.00	0.99; 1.01	0.626	1.00	1.00; 1.01	0.530	1.01	0.99; 1.03	0.222
Social anhedonia	Smoking initiation	1.00	0.99; 1.00	0.746	1.00	0.99; 1.00	0.754	1.00	1.00; 1.01	0.294	1.00	1.00; 1.01	0.578	1.00	0.99; 1.02	0.523
Paranoia/hallucinations	Smoking initiation	1.00	0.99; 1.01	0.880	1.00	0.99; 1.01	0.720	0.99	0.96; 1.01	0.330	0.99	0.98; 1.01	0.461	0.99	0.96; 1.01	0.331
Cognitive disorganisation	Smoking initiation	1.01	1.00; 1.02	0.190	1.00	1.00; 1.01	0.307	0.98	0.96; 1.00	0.059	1.00	0.99; 1.01	0.720	1.00	0.98; 1.02	0.945
Anhedonia	Smoking initiation	1.00	0.99; 1.01	0.533	1.00	0.99; 1.01	0.599	1.00	0.98; 1.02	0.965	1.00	0.99; 1.01	0.944	1.00	0.98; 1.02	0.864
Negative symptoms	Smoking initiation	1.00	0.99; 1.01	0.712	1.00	0.99; 1.01	0.609	0.99	0.96; 1.02	0.436	1.00	0.98; 1.01	0.656	1.00	0.98; 1.03	0.770

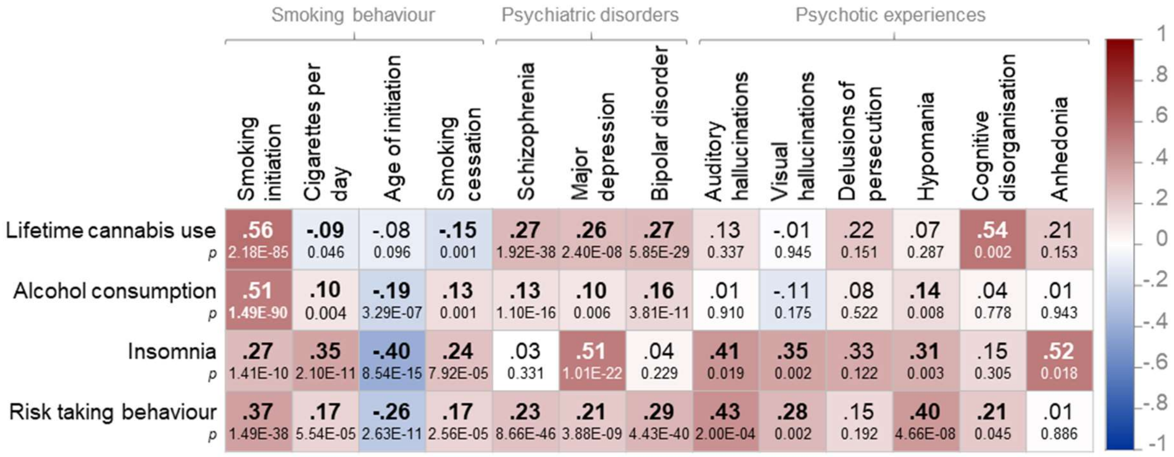

Supplementary Figure S1. Genetic correlations between smoking behaviours, psychotic experiences and psychiatric disorders with covariates used in genomic multiple regression

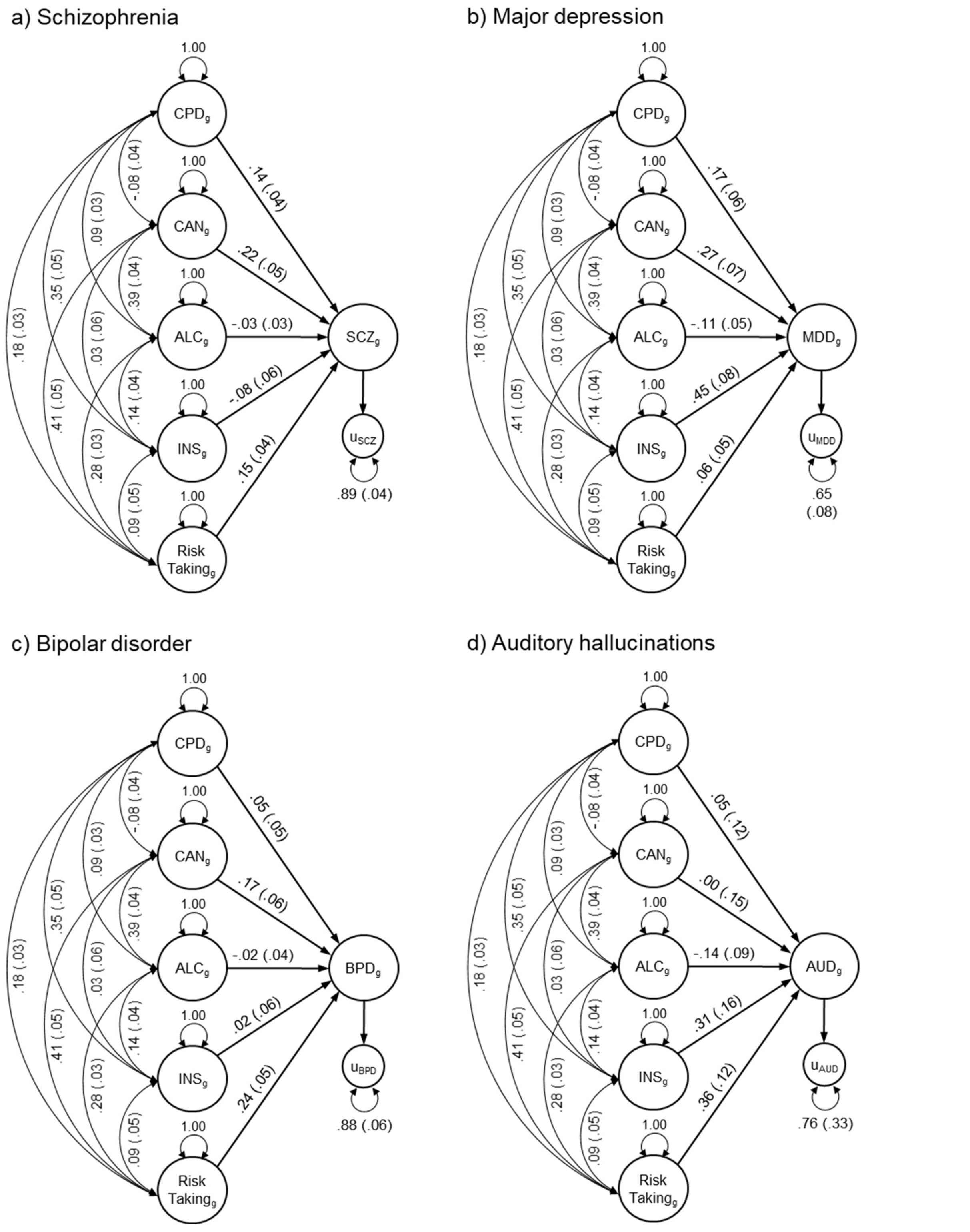

Supplementary Figure S2. Genomic multiple regression models for cigarettes per day as a predictor of psychiatric disorders and psychotic experiences

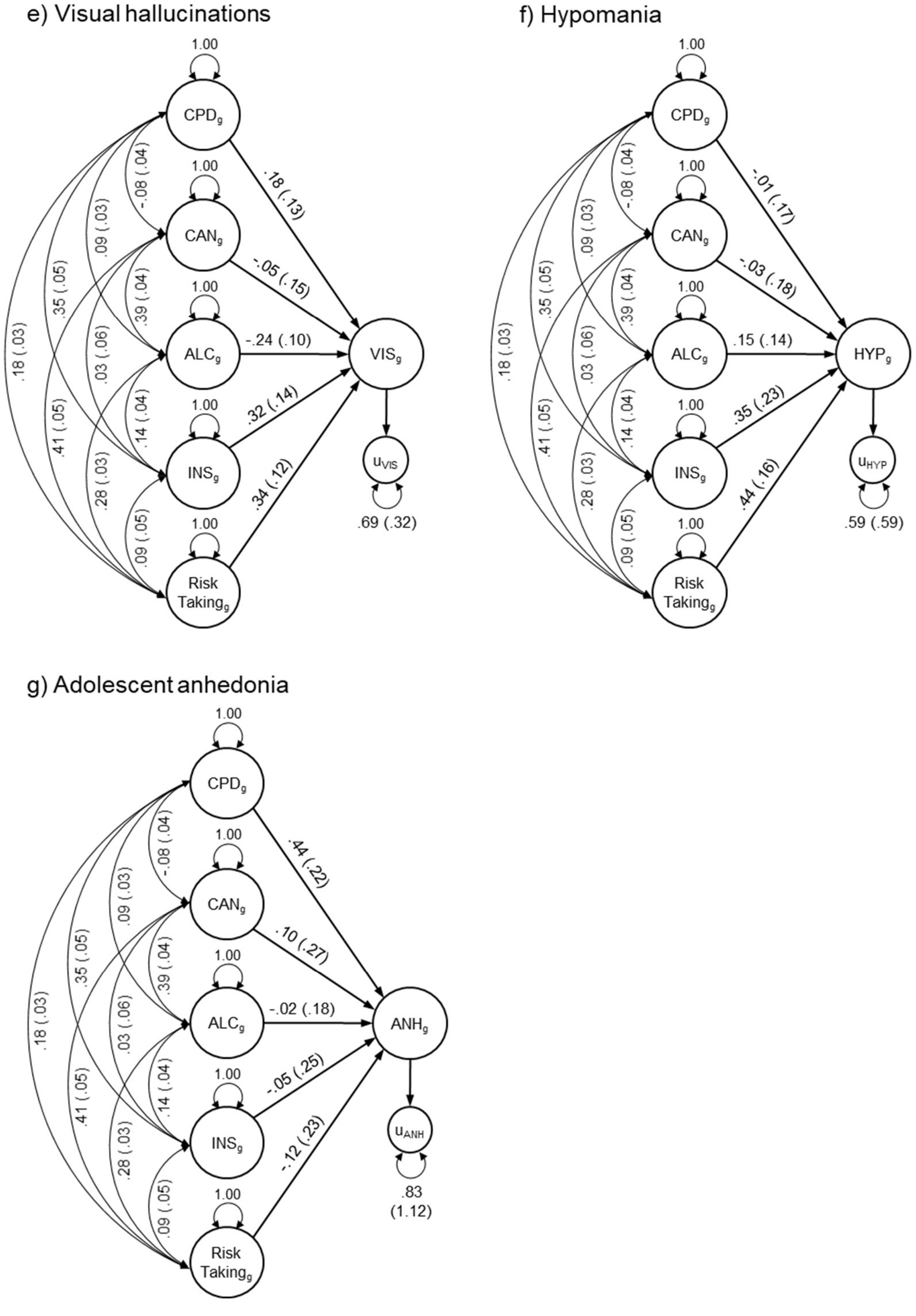

Supplementary Figure S2. (continued)

Circles indicate genome-wide genetic influences on cigarettes per day (CPDg), lifetime cannabis use (CANg), alcohol consumption (ALCg), insomnia (INSg), schizophrenia (SCZg), major depressive disorder (MDDg), bipolar disorder (BPDg), auditory hallucinations (AUDg), visual hallucinations (VISg), hypomania (HYPg) and adolescent anhedonia (ANHg); Double headed arrows on the left of the figures display the genetic correlations between phenotypes. Single-headed arrows on the right of each figure represent the regression of the genetic predictors on the genetic components of the outcomes. The conditional genetic associations are displayed beside arrow lines with standard errors in parentheses; U = residual variance.

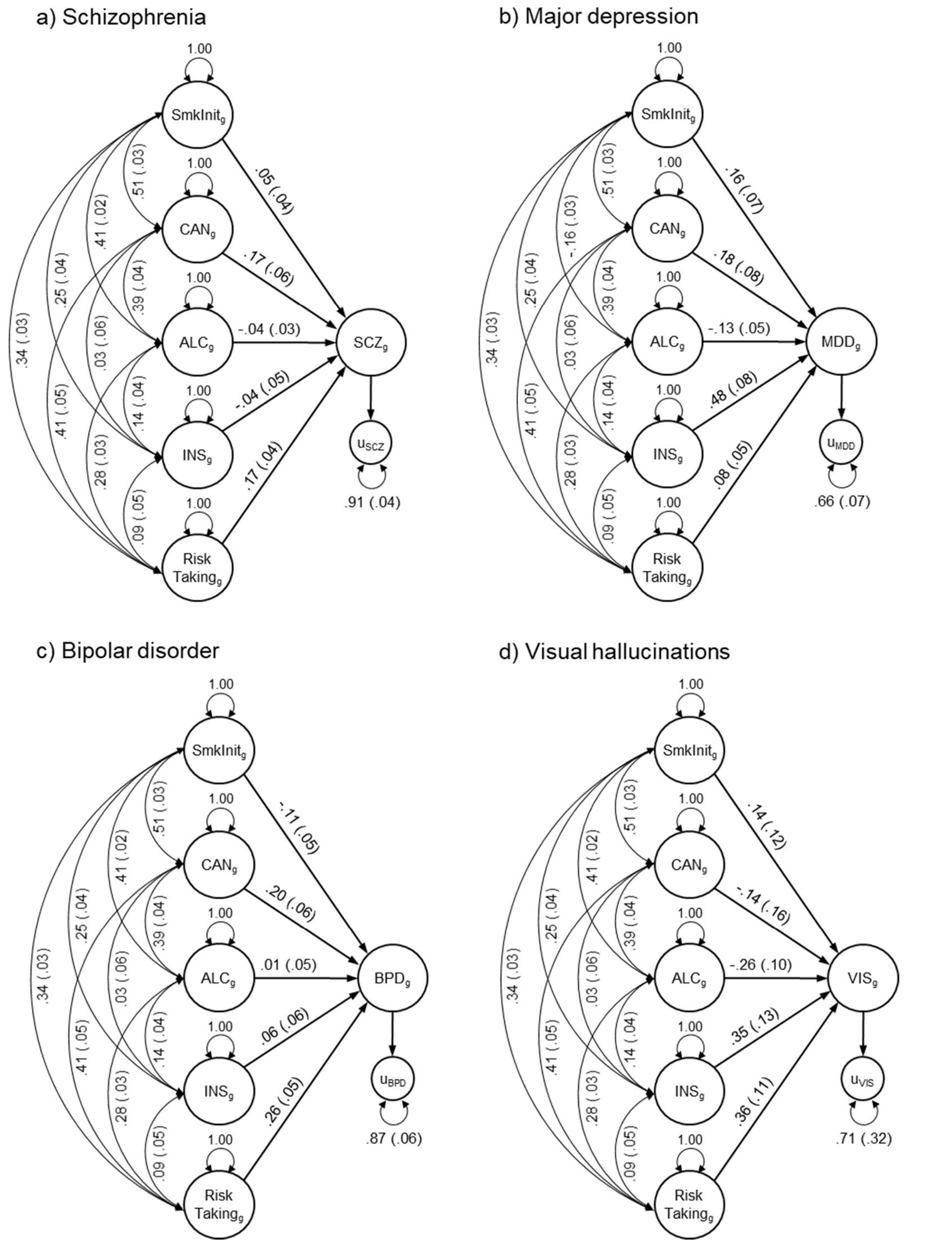

Supplementary Figure S3. Genomic multiple regression models for smoking initiation as a predictor of psychiatric disorders and psychotic experiences

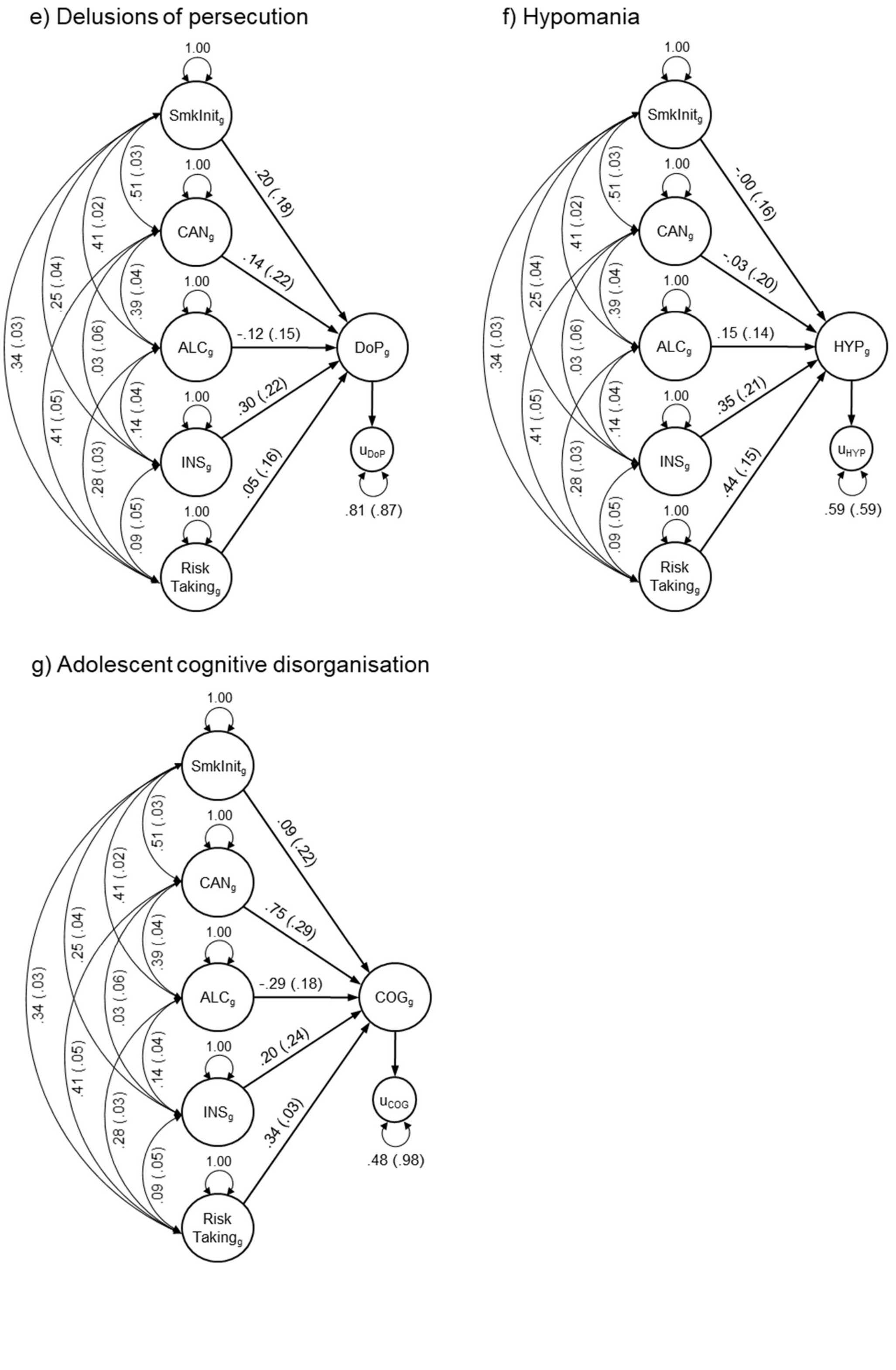

Supplementary Figure S3. (continued)

Circles indicate genome-wide genetic influences on smoking initiation ($SmkInit_g$), lifetime cannabis use (CANg), alcohol consumption (ALCg), insomnia (INSg), schizophrenia (SCZg), major depressive disorder (MDDg), bipolar disorder (BPDg), visual hallucinations (VISg), delusions of persecution (DoPg), hypomania (HYPg) and adolescent cognitive disorganization (COGg); Double headed arrows on the left of the figures display the genetic correlations between phenotypes. Single-headed arrows on the right of each figure represent the regression of the genetic predictors on the genetic components of the outcomes. The conditional genetic associations are displayed beside arrow lines with standard errors in parentheses; U = residual variance.

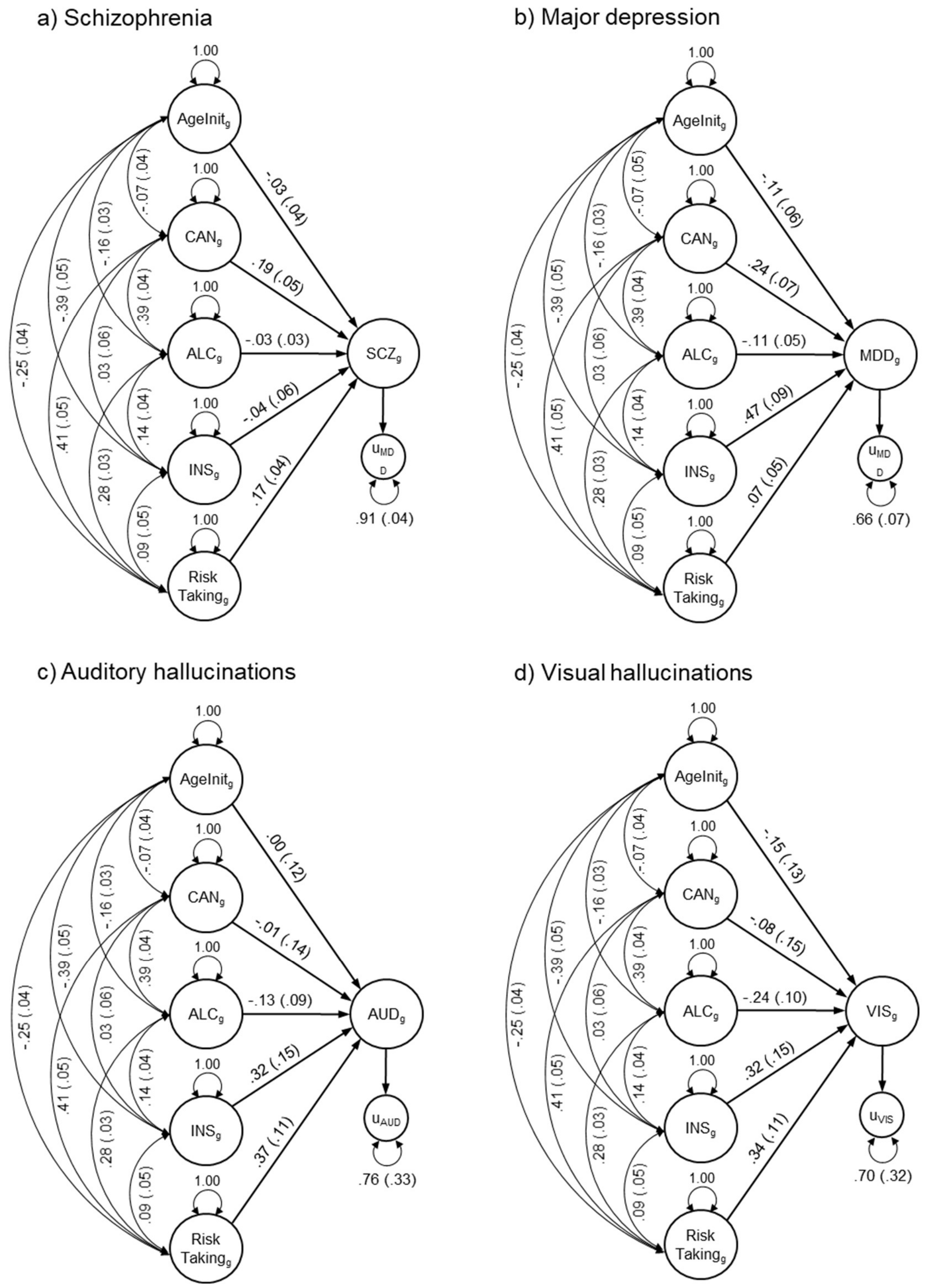

Supplementary Figure S4. Genomic multiple regression models for age of smoking initiation as a predictor of psychiatric disorders and psychotic experiences

e) Hypomania

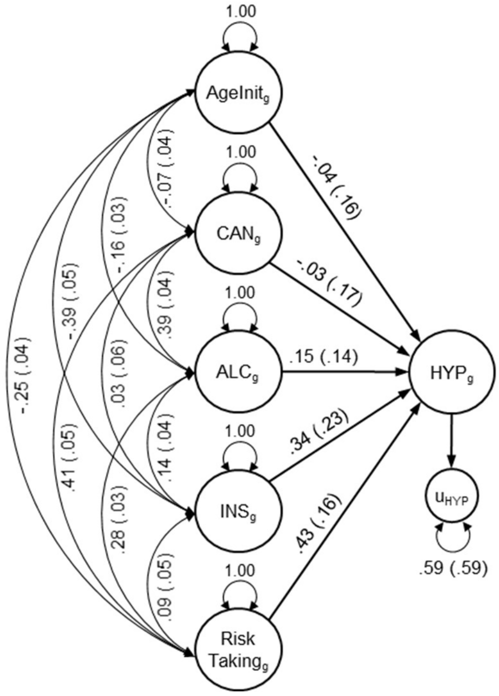

Supplementary Figure S5. (continued)

Circles indicate genome-wide genetic influences on age of smoking initiation (AgeInitg), lifetime cannabis use (CANg), alcohol consumption (ALCg), insomnia (INSg), schizophrenia (SCZg), major depressive disorder (MDDg), auditory hallucinations (AUDg), visual hallucinations (VISg) and hypomania (HYPg); Double headed arrows on the left of the figures display the genetic correlations between phenotypes. Single-headed arrows on the right of each figure represent the regression of the genetic predictors on the genetic components of the outcomes. The conditional genetic associations are displayed beside arrow lines with standard errors in parentheses; U = residual variance.

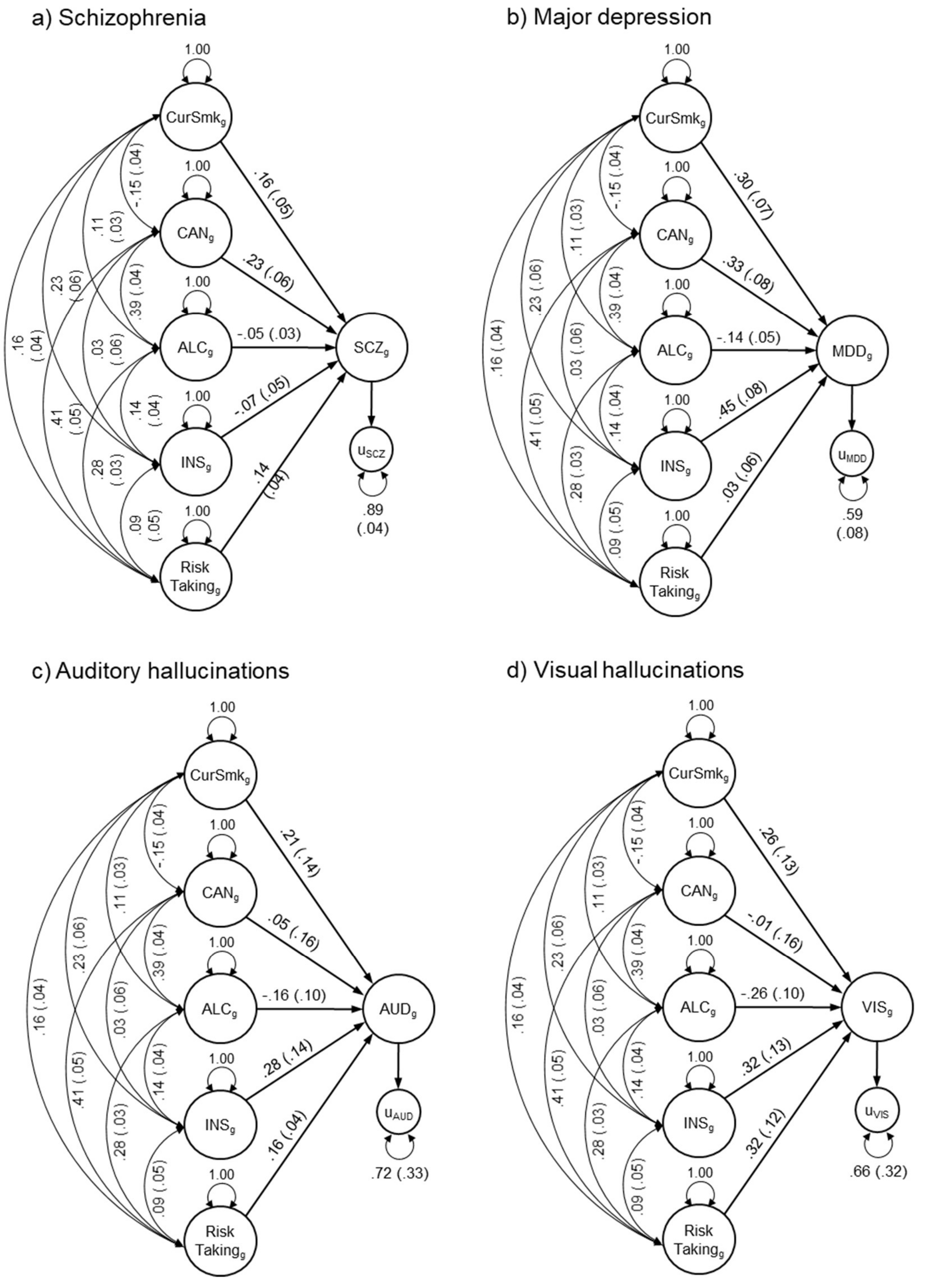

Supplementary Figure S6. Genomic multiple regression models for current smoking status as a predictor of psychiatric disorders and psychotic experiences

Circles indicate genome-wide genetic influences on age of current smoking status (CurSmkg), lifetime cannabis use (CANg), alcohol consumption (ALCg), insomnia (INSg), schizophrenia (SCZg), major depressive disorder (MDDg), auditory hallucinations (AUDg) and visual hallucinations (VISg); Double headed arrows on the left of the figures display the genetic correlations between phenotypes. Single-headed arrows on the right of each figure represent the regression of the genetic predictors on the genetic components of the outcomes. The conditional genetic associations are displayed beside arrow lines with standard errors in parentheses; U = residual variance.

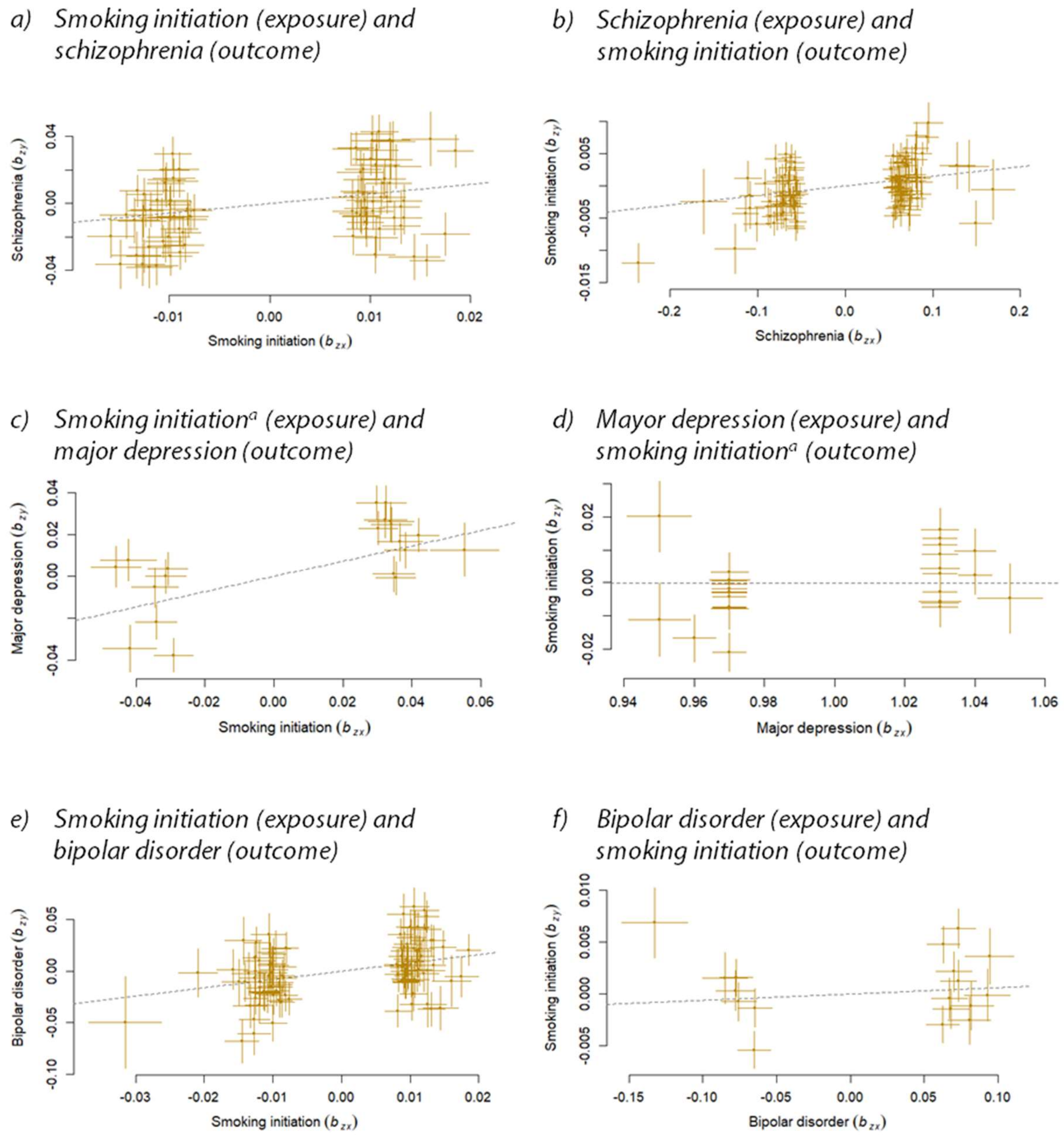

Supplementary Figure S7. Generalised Summary-Based Mendelian Randomization analyses between psychiatric disorders and smoking initiation

Scatterplots with the x-axis displaying instrumental variable effects on the exposure (b_{zx}) and the y-axis displaying the instrument-outcome association (b_{zy}). Regression lines included for reference.

[a] *For analyses on major depression, summary statistics for smoking initiation excluded UK Biobank participants to avoid overlapping samples (N = 249,171).*

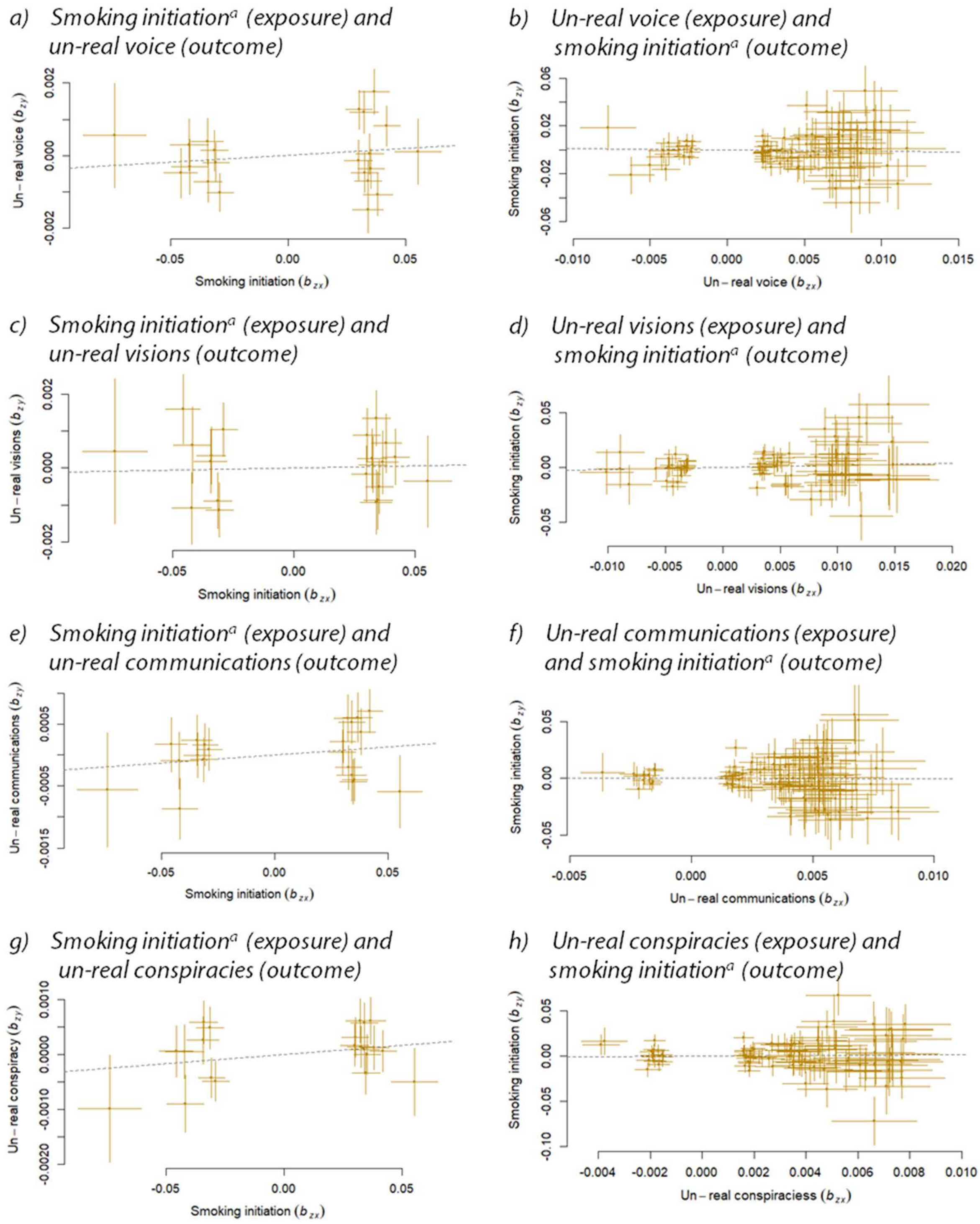

Supplementary Figure S8. Generalised Summary-Based Mendelian Randomization between positive psychotic experiences in adulthood and smoking initiation

Scatterplots with the x-axis displaying instrumental variable effects on the exposure (b_{zx}) and the y-axis displaying the instrument-outcome association (b_{zy}). Regression lines included for reference.

[a] For analyses on adult psychotic experiences, summary statistics for smoking initiation excluded UK Biobank participants to avoid overlapping samples (N = 249,171).

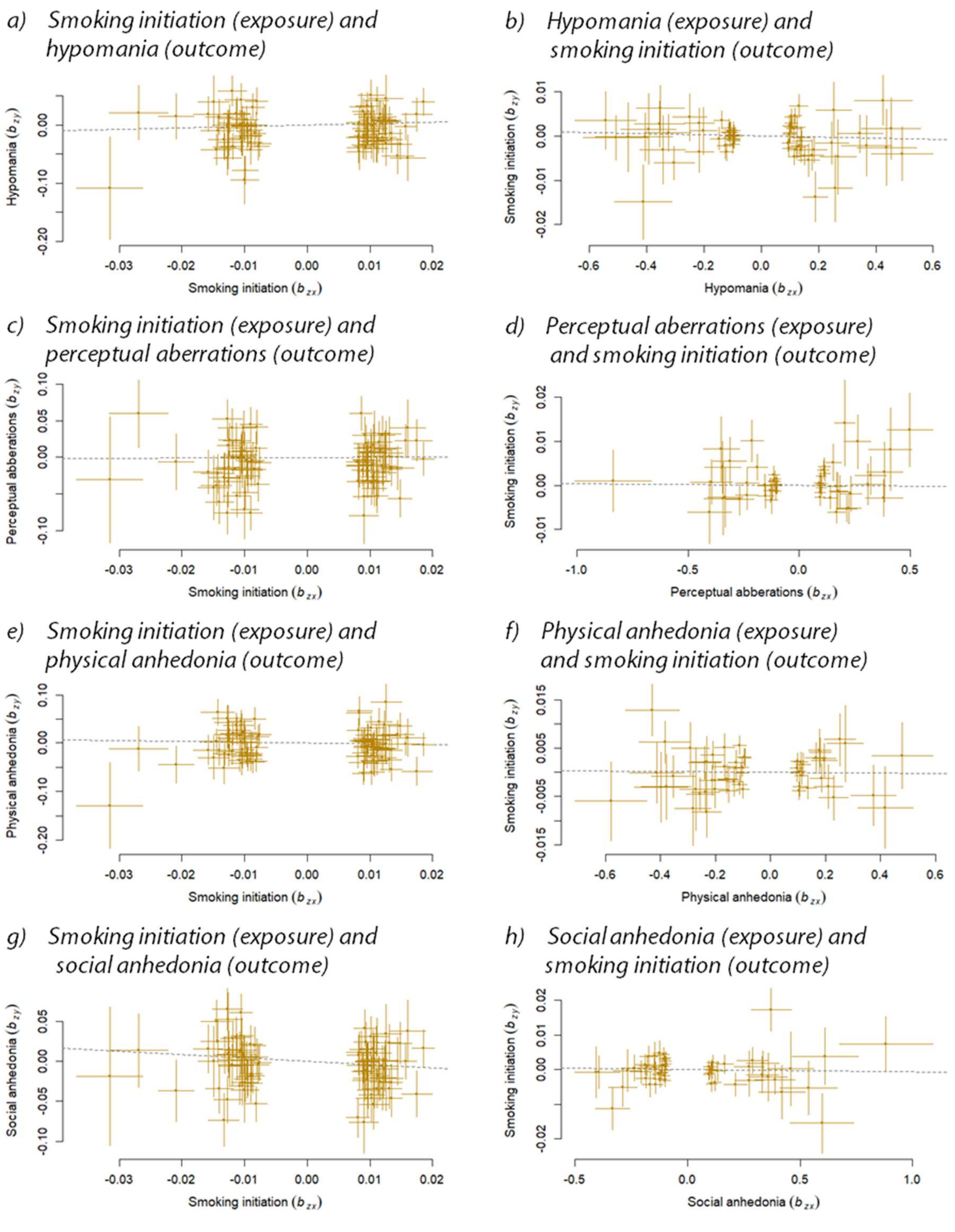

Supplementary Figure S9. Generalised Summary-Based Mendelian Randomization between schizotypy in adulthood and smoking initiation

Scatterplots with the x-axis displaying instrumental variable effects on the exposure (b_{zx}) and the y-axis displaying the instrument-outcome association (b_{zy}). Regression lines included for reference.

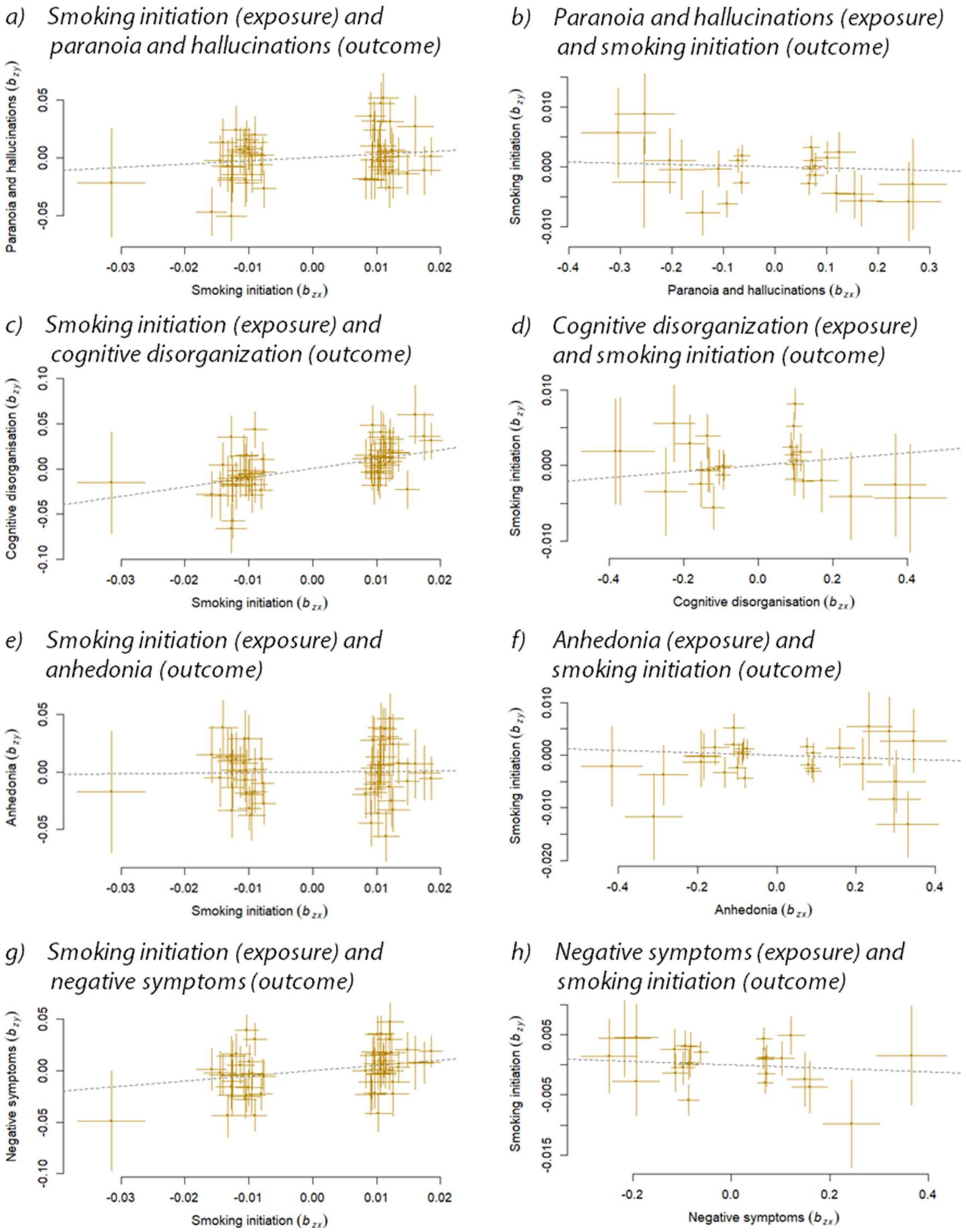

Figure S10. Generalised Summary-Based Mendelian Randomization between adolescent psychotic experiences and negative symptom traits and smoking initiation

Scatterplots with the x-axis displaying instrumental variable effects on the exposure (b_{zx}) and the y-axis displaying the instrument-outcome association (b_{zy}). Regression lines included for reference.

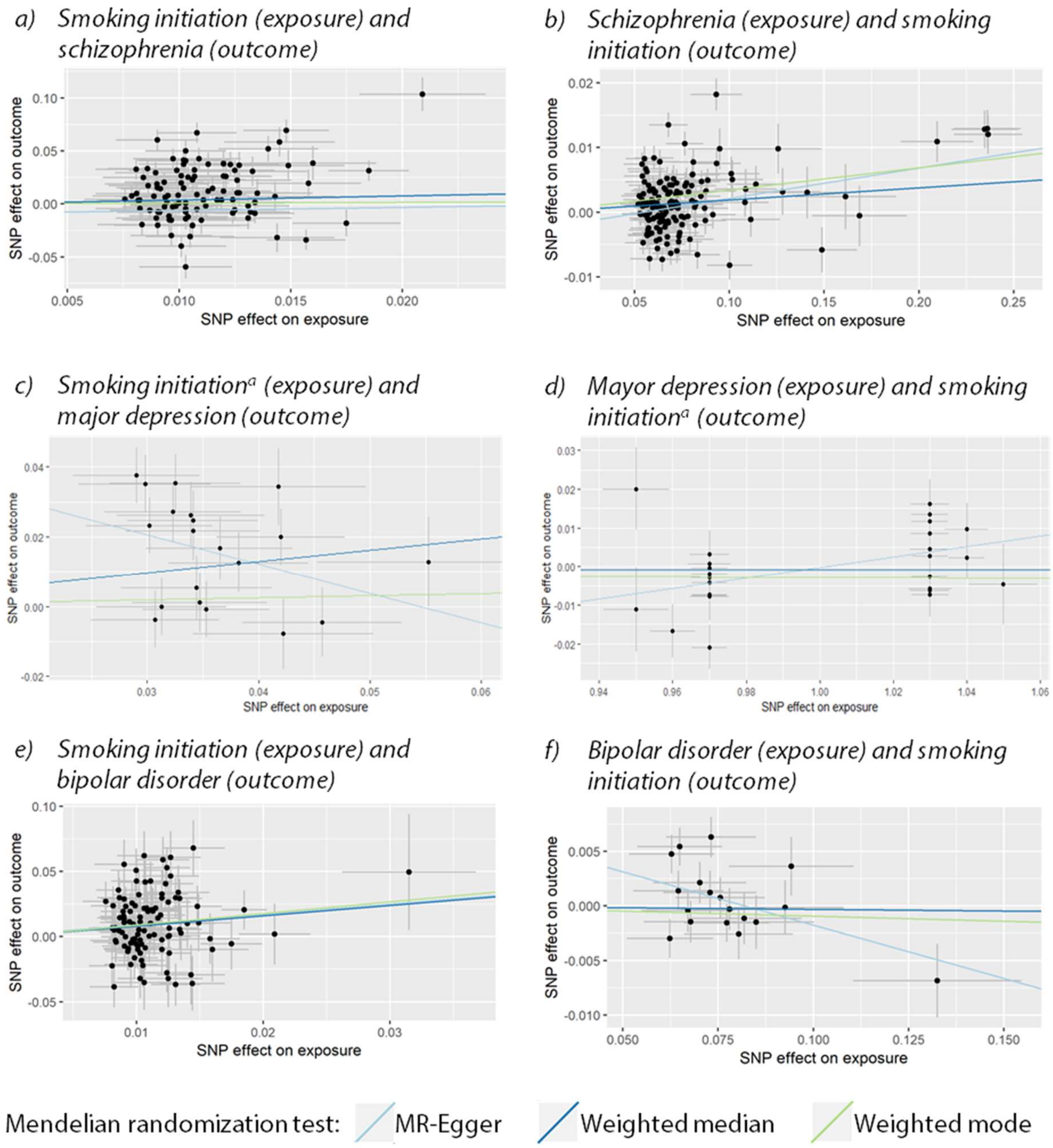

Supplementary Figure S11. MR-Egger, Weighted Median and Weighted Mode Mendelian randomization sensitivity analyses between psychiatric disorders and smoking initiation

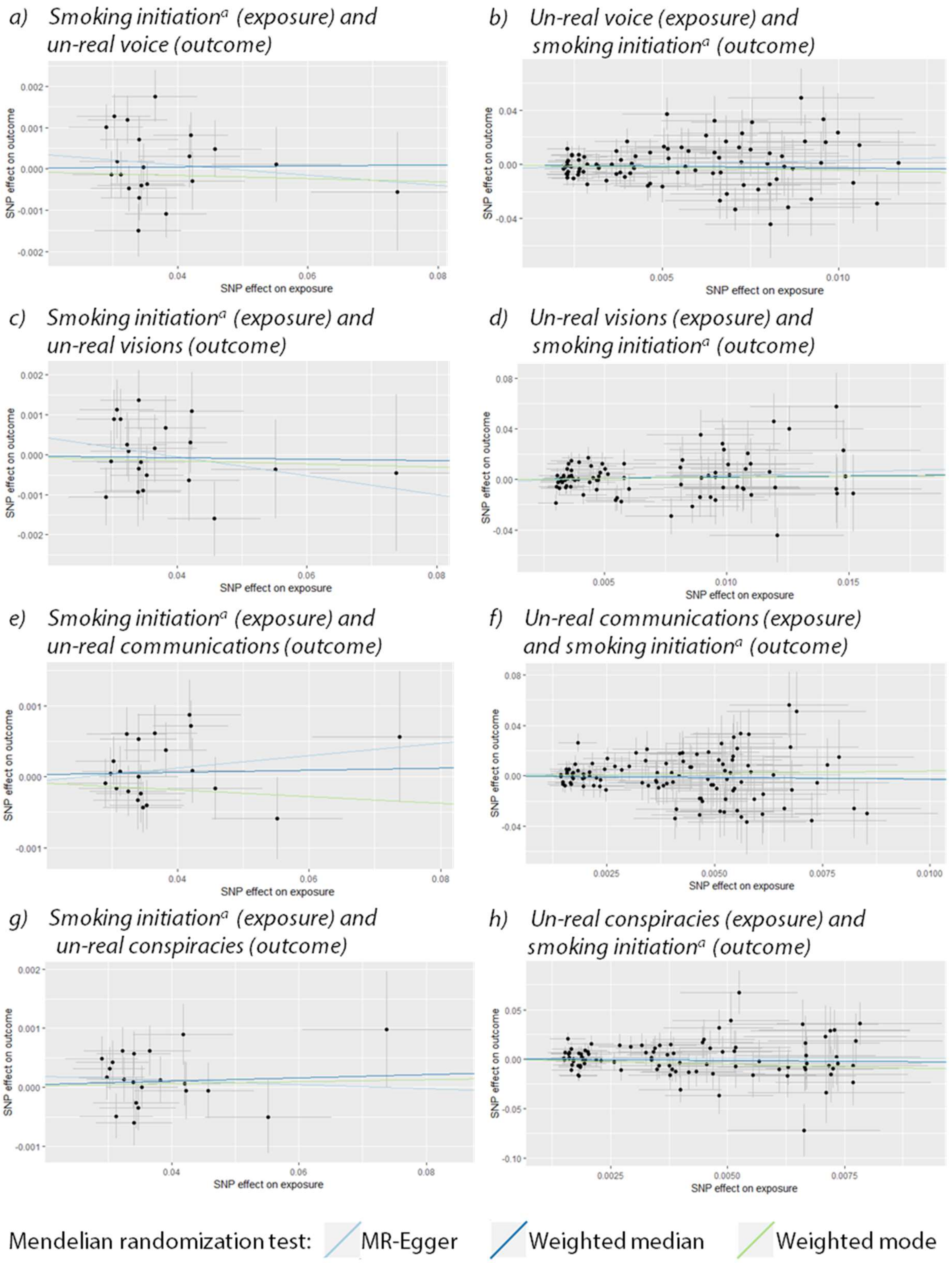

Supplementary Figure S12. MR-Egger, Weighted Median and Weighted Mode Mendelian randomization sensitivity analyses between positive psychotic experiences in adulthood and smoking initiation

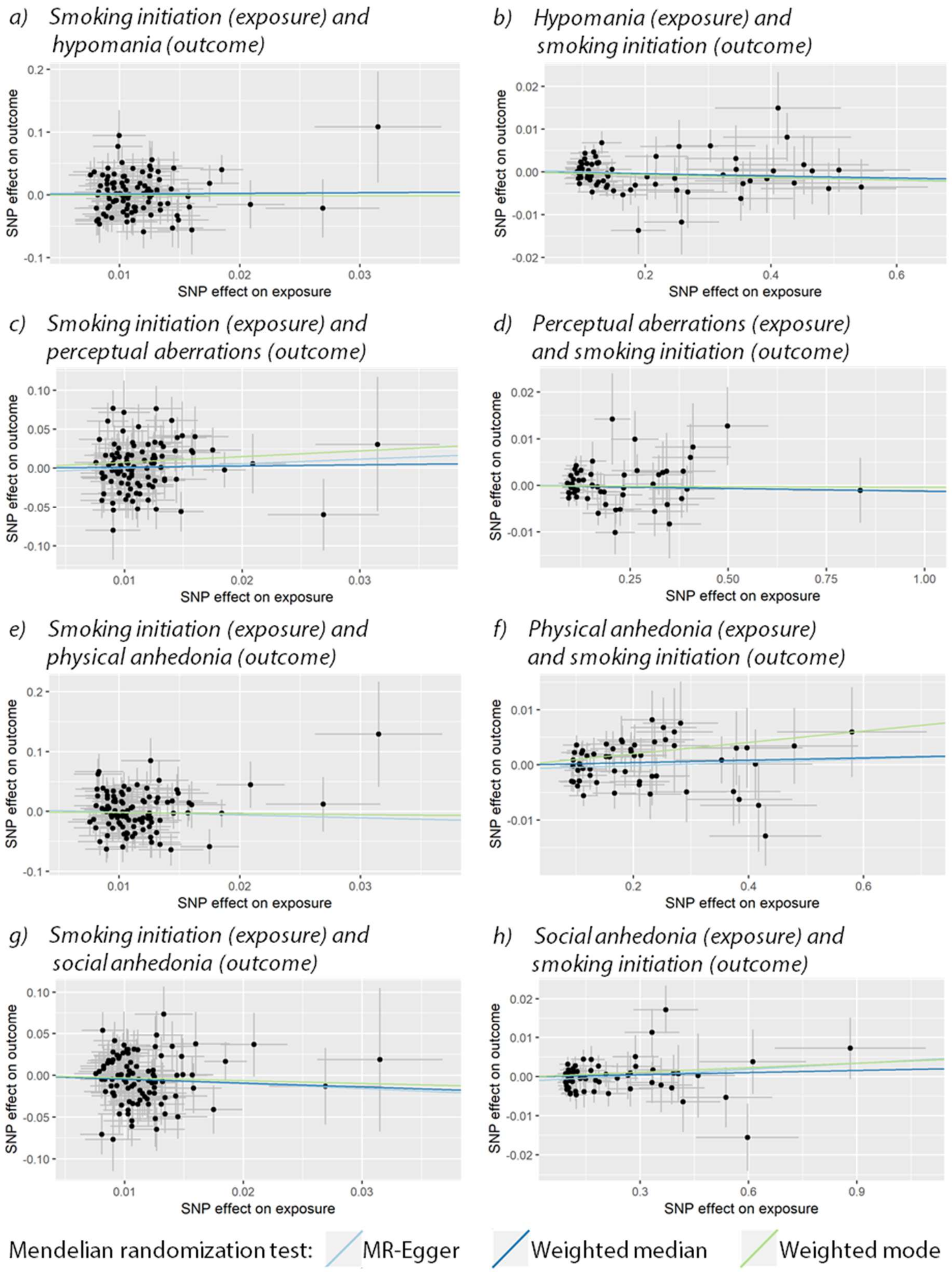

Supplementary Figure S13. MR-Egger, Weighted Median and Weighted Mode Mendelian randomization sensitivity analyses between schizotypy in adulthood and smoking initiation

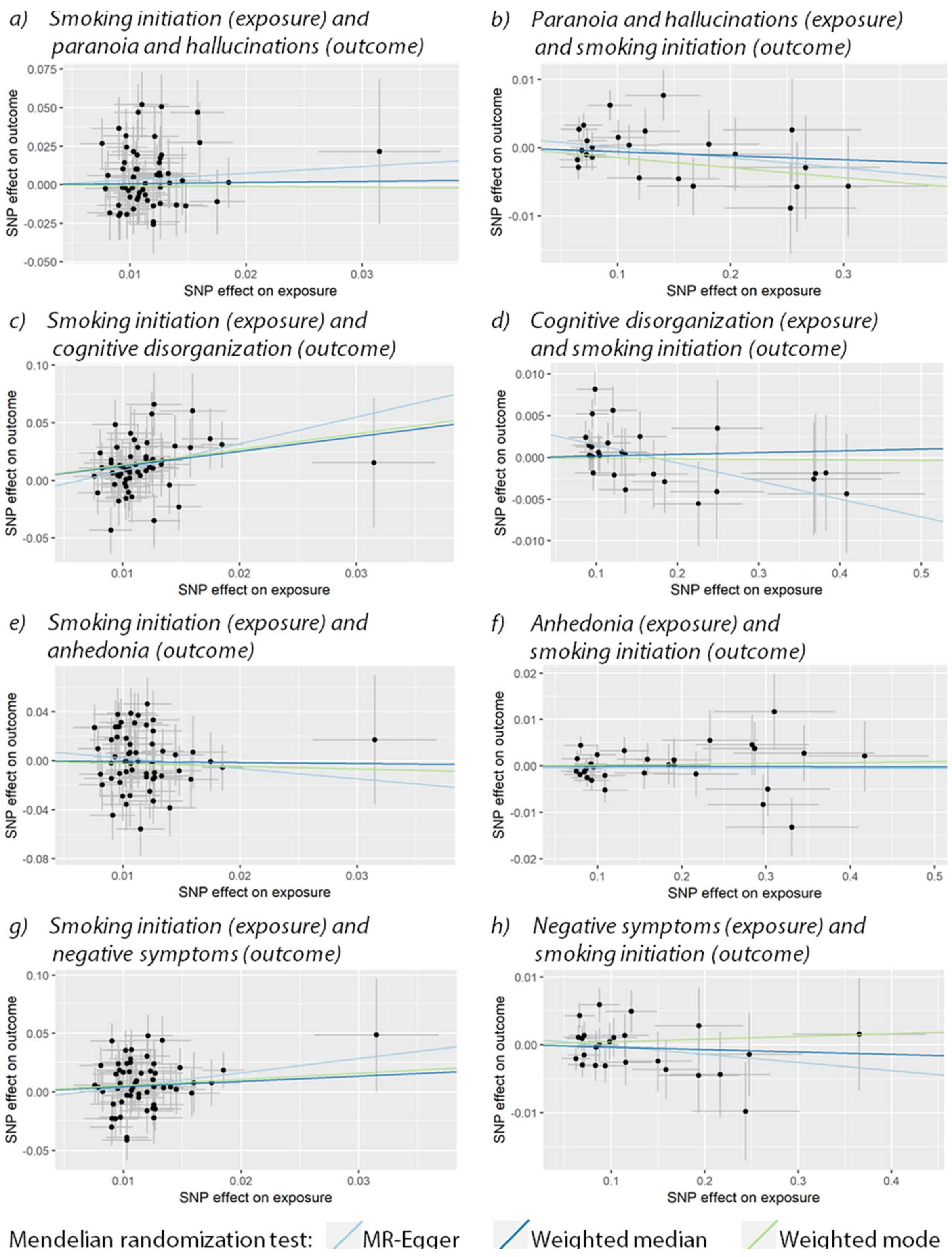

Supplementary Figure S14. MR-Egger, Weighted Median and Weighted Mode Mendelian randomization sensitivity analyses between adolescent psychotic experiences and negative symptom traits and smoking initiation

Supplementary Methods

Samples and measures

Adolescent psychotic experiences and negative symptoms

Summary statistics were obtained from a mega-GWAS of four continuous scales of adolescent PENS among participants of European ancestry: Paranoia and hallucinations, cognitive disorganisation, parent-rated negative symptoms and anhedonia (N 6,297-10,098).[2] PENS items came from three community-based samples: The Twins Early Development Study (TEDS)[7] had a mean age of 16.32 years at the time of assessment, the Avon Longitudinal Study of Parents and Children (ALSPAC; mean age 16.76 years),[8,9] and the Child and Adolescent Twin Study in Sweden (CATSS; mean age 18.31 years).[10]

The ALSPAC sample: Pregnant women resident in Avon, UK and with an expected delivery date between 1st April 1991 and 31st December 1992 was invited to participate in the ALSPAC study. The initial sample consisted of 14,775 children. Informed consent for the use of data collected via questionnaires and clinics was obtained from participants following the recommendations of the ALSPAC Ethics and Law Committee at the time. Consent for biological samples was collected in accordance with the Human Tissue Act (2004). The number of genotyped individuals who completed items on psychotic experiences (after exclusions) was 3,951 – 4,019. The ALSPAC study website contains details of all the data that is available through a fully searchable data dictionary and variable search tool (http://www.bristol.ac.uk/alspac/researchers/our-data/).

Schizotypy in adulthood

GWAS on four continuous schizotypy scales assessed during middle adulthood in the Northern Finland Birth Cohort 1996 (NFBC)[11] when participants were aged 31 years were obtained from the authors (N 3,967 – 4.057).[3] Perceptual aberrations were assessed using the Perceptual Aberration Scale,[12] hypomania using the Hypomanic Personality Scale,[13] social anhedonia with the Revised Social Anhedonia Scale and physical anhedonia using the Revised Physical Anhedonia Scale.[14]

Psychotic experiences in adults

The presence of lifetime positive PE were assessed in the UK Biobank using four dichotomous items as part of a mental health questionnaire completed by 157,397 participants aged 40-69 years. Participants reported an average age of PE onset of 31.6 (s.d. = 17.6) years. Summary statistics for individuals of European ancestry were obtained from the Neale Lab (http://www.nealelab.is/uk-biobank) on experiences of auditory hallucinations, visual hallucinations, delusions of persecution and delusions of reference.

Psychiatric disorders

Summary statistics were obtained from the Psychiatric Genomics Consortium (https://www.med.unc.edu/pgc/results-and-downloads) meta-GWAS for schizophrenia[4] (N= 105,318), major depressive disorder[6] (N = 173,005 excluding 23andMe participants) and bipolar disorder[5] (N = 41,653). Diagnosis of schizophrenia was based on DSM-IV criteria for schizophrenia or schizoaffective disorder. Major depression diagnoses were based on clinical interviews, obtained from electronic healthcare records or based on self-report in some UK Biobank participants. Different clinical interview formats were used to diagnose Bipolar disorder, described in full elsewhere.[5]

Covariates in genomic multiple regression

Publicly available summary statistics were obtained for lifetime cannabis use (N = 162,082),[15] alcohol consumption (N = 537,349 excluding 23andMe participants),[1] risk taking (Neale Lab; N = 348,549 UK Biobank participants) and insomnia (N= 113,006).[16] Cannabis use was a binary phenotype assessed using self-report items on whether participants had ever used cannabis. Alcohol consumption came from participant reports on the average number of weekly drinks they drank. Risk taking was assessed with the item “Would you describe yourself as someone who takes risks?” (UK Biobank data-field 2040). Insomnia was from an item from the UK Biobank (data-field 1200) with participants who indicated that they usually have trouble falling asleep at night or wake up in the middle of the night classed as cases.

Mendelian randomization

Mendelian randomization (MR) is a method used to test for a causal relationship between an exposure and outcome trait by using instrumental variables as proxies for the exposure trait. In MR, genetic variants that are robustly associated with the exposure (based on GWAS results) are used as instrumental variables. The random nature of Mendelian segregation of genetic variants during meiosis means that the extent to which unmeasured confounders influence the outcome is not expected to differ between those who inherited a specific copy of a genetic variant and those who did not (analogous to randomization during randomized controlled trials).

In MR, a causal effect of an exposure (X) on an outcome (Y) is calculated as the ratio of the effect size of an instrumental variable (Z) on the outcome over its effect on the exposure: $\hat{b}_{XY} = \hat{b}_{ZY}/\hat{b}_{ZX}$, where $\hat{b}_{XY}$ is the causal effect of the exposure on the outcome, $\hat{b}_{ZY}$ is the effect of the instrumental variable on the outcome and $\hat{b}_{ZX}$ is its effect on the exposure. To overcome the small effect sizes of individual genetic variants, an aggregate $\hat{b}_{XY}$ effect can be obtained using multiple variants as instrumental variables.

References

1 Liu, M. *et al*. Association studies of up to 1.2 million individuals yield new insights into the genetic etiology of tobacco and alcohol use. *Nat. Genet*. **51**, 237-244, doi:10.1038/s41588-018-0307-5 (2019).

2 Pain, O. *et al*. Genome-wide analysis of adolescent psychotic-like experiences shows genetic overlap with psychiatric disorders. *Am. J. Med. Genet. B Neuropsychiatr. Genet.* **177**, 416-425, doi:10.1002/ajmg.b.32630 (2018).

3 Ortega-Alonso, A. *et al*. Genome-Wide Association Study of Psychosis Proneness in the Finnish Population. *Schizophr. Bull.* **43**, 1304-1314, doi:10.1093/schbul/sbx006 (2017).

4 Pardinas, A. F. *et al*. Common schizophrenia alleles are enriched in mutation-intolerant genes and in regions under strong background selection. *Nat. Genet.* **50**, 381-389, doi:10.1038/s41588-018-0059-2 (2018).

5 Bipolar Disorder and Schizophrenia Working Group of the Psychiatric Genomics Consortium. Genomic Dissection of Bipolar Disorder and Schizophrenia, Including 28 Subphenotypes. *Cell* **173**, 1705-1715.e1716, doi:10.1016/j.cell.2018.05.046 (2018).

6 Wray, N. R. *et al*. Genome-wide association analyses identify 44 risk variants and refine the genetic architecture of major depression. *Nat. Genet.* **50**, 668-681, doi:10.1038/s41588-018-0090-3 (2018).

7 Haworth, C. M., Davis, O. S. & Plomin, R. Twins Early Development Study (TEDS): a genetically sensitive investigation of cognitive and behavioral development from childhood to young adulthood. *Twin Res. Hum. Genet.* **16**, 117-125, doi:10.1017/thg.2012.91 (2013).

8 Boyd, A. *et al*. Cohort Profile: the 'children of the 90s'--the index offspring of the Avon Longitudinal Study of Parents and Children. *Int. J. Epidemiol.* **42**, 111-127, doi:10.1093/ije/dys064 (2013).

9 Fraser, A. *et al*. Cohort Profile: the Avon Longitudinal Study of Parents and Children: ALSPAC mothers cohort. *Int. J. Epidemiol*. **42**, 97-110, doi:10.1093/ije/dys066 (2013).

10 Anckarsater, H. *et al*. The Child and Adolescent Twin Study in Sweden (CATSS). *Twin Res. Hum. Genet.* **14**, 495-508 (2011).

11 Haapea, M. *et al*. Non-participation in a field survey with respect to psychiatric disorders. *Scand. J. Public Health* **36**, 728-736, doi:10.1177/1403494808092250 (2008).

12 Chapman, L. J., Chapman, J. P. & Raulin, M. L. Body-image aberration in Schizophrenia. *J. Abnorm. Psychol.* **87**, 399-407 (1978).

13 Eckblad, M. & Chapman, L. J. Development and validation of a scale for hypomanic personality. *J. Abnorm. Psychol*. **95**, 214-222 (1986).

14 Chapman, L. J., Chapman, J. P. & Raulin, M. L. Scales for physical and social anhedonia. *J. Abnorm. Psychol.* **85**, 374-382 (1976).

15 Pasman, J. A. *et al*. GWAS of lifetime cannabis use reveals new risk loci, genetic overlap with psychiatric traits, and a causal influence of schizophrenia. *Nat. Neurosci*. **21**, 1161-1170, doi:10.1038/s41593-018-0206-1 (2018).

16 Hammerschlag, A. R. *et al*. Genome-wide association analysis of insomnia complaints identifies risk genes and genetic overlap with psychiatric and metabolic traits. *Nat. Genet.* **49**, 1584-1592, doi:10.1038/ng.3888 (2017).
